# Urban planning effectiveness and citizen satisfaction. A systematic literature review

**DOI:** 10.12688/f1000research.157550.1

**Published:** 2024-11-06

**Authors:** Yefferson Llonto Caicedo, Rogger Orlando Morán Santamaría, Guido Alarcón Villanueva, Leticia Noemi Zavaleta Gonzáles, Willy Darwin Llatas Díaz, Ida Blanca Pacheco Gonzales, Rocío Janet Pejerrey González, Percy Junior Castro Mejía, Carlos William Atalaya Urrutia

**Affiliations:** 1 Lambayeque, Universidad Nacional Pedro Ruiz Gallo, Lambayeque, Lambayeque, Peru; 2 La Libertad, Universidad Cesar Vallejo, Trujillo, La Libertad, Peru; 3 Cajamarca, Universidad Nacional de Cajamarca, Cajamarca, Cajamarca, Peru; 4 Lambayeque, Universidad Senor de Sipan, Chiclayo, Lambayeque, Peru

**Keywords:** PRISMA, bibliometrics, bibliometrix, urban planning, citizen satisfaction, systematic review, state of the art.

## Abstract

**Background:**

The population is increasingly demanding a better quality of life in their territories, which requires better urban planning. This study aims to find out the effectiveness of urban planning implemented by local governments on citizen satisfaction.

**Method:**

A systematic literature review was conducted exploring the evolution of the state-of-the-art databases in Scopus, WOS and Dimensions, involving a relevant selection of empirical studies on the effectiveness of urban planning on citizen satisfaction, using quality criteria and the application of the PRISMA diagram.

**Results:**

The findings in the various empirical contributions find converging in three main blocks the contributions related to: (i) Urban planning as a catalyst for the impact of citizen satisfaction, given that using the planning tool will lead to the development of local policies based on the Neoliberalism approach for smart urban development; (ii) Theoretical contributions for urban planning that contextualises a modernist planning based on a multidimensional aspect to address quality of life for modern well-being and (iii) Smart planning for citizen satisfaction for the design and implementation of public governance reforms aimed at optimising urban planning management at the local level for smart urban city development.

**Conclusion:**

There is strong theoretical and empirical support that is closely linked to sustainable development, happiness, public space, urban growth, urban areas, satisfied customers and urban residents. Future research should examine the relative weight of urban planning dimensions and effectiveness in the sustainable development of territories and citizen satisfaction.

## Introduction

In recent decades, urban planning has acquired a heightened level of relevance due to the phenomenon of urbanisation and the associated challenges that it presents, including those pertaining to environmental sustainability, urban mobility and the quality of life of citizens ( Bhuiyan & Islam, 2023; Sanchez et al., 2024; Canal et al., 2023). The effectiveness of urban planning has become a crucial issue to ensure more liveable and resilient cities. Globally, efforts have been directed towards integrating advanced technologies and innovative concepts, such as smart cities, which aim to optimise the management of urban resources and improve resident satisfaction ( Supangkat et al., 2023; Papageorgiou et al., 2024; Alfaro-Navarro et al., 2024). Nevertheless, the implementation of these plans does not always result in the improvements that citizens perceive, which gives rise to questions about the relationship between urban planning and citizen satisfaction ( Lim et al., 2019).

In particular, citizen satisfaction has emerged as a pivotal metric for evaluating the efficacy of urban interventions ( Wang et al., 2023). A number of studies have examined the impact of infrastructure, public services and sustainability initiatives on the perception and well-being of inhabitants. However, the results are variable and frequently contingent on the specific context of each city. This lack of consensus in the literature reflects the complexity of urban dynamics and the necessity for more holistic and personalised approaches that consider the expectations and needs of communities.

Furthermore, research has demonstrated that the incorporation of green spaces and recreational areas into urban planning strategies has a considerable positive effect on citizen satisfaction. Such spaces not only enhance the visual appeal of urban environments but also confer tangible benefits for the mental and physical well-being of residents ( Rico et al., 2022).

The quality of infrastructure and the availability of public services, such as transport, education and health, are pivotal factors in determining levels of citizen satisfaction. The implementation of enhanced transport infrastructure has been demonstrated to diminish travel times and, moreover, to enhance the general perception of governmental efficiency.

Urban planning represents a crucial instrument for local governments in their pursuit of enhancing the quality of life of their citizens. The integration of elements of sustainability, participation and infrastructure development has been demonstrated to be the most effective strategy for increasing citizen satisfaction. It is recommended that future studies adopt an interdisciplinary approach combining quantitative and qualitative data in order to gain a deeper understanding of the factors influencing urban satisfaction.

Notwithstanding the endeavours to enhance the quality of urban life, there remain deficiencies in our comprehension of the manner in which diverse urban planning strategies impact citizen satisfaction. Recent studies have indicated that while certain urban planning strategies may improve specific aspects of urban life, such as mobility or access to services, there is not always a direct correlation with increased overall resident satisfaction. This is due to the fact that the effectiveness of such planning strategies is currently unknown, given the numerous constraints faced by local governments and the weak management capacities that often result in ineffectiveness ( Guillen et al., 2017; Supangkat et al., 2023).

The objective of this study is to conduct a comprehensive review of the existing literature on the effectiveness of urban planning implemented by local governments in influencing citizen satisfaction. This is a crucial issue as citizen satisfaction directly impacts the quality of life of citizens, economic development and environmental sustainability.

The theoretical contribution of the currents suggests that the effectiveness of urban planning is influenced by a number of theoretical and doctrinal factors, including the adoption of participatory approaches, the integration of smart technologies and the incorporation of social equity considerations in decision-making processes ( Sohail et al., 2005; Wu et al., 2022).

The field of urban planning has undergone significant evolution over the past few decades, giving rise to a multitude of doctrinal approaches that seek to address the complex challenges facing contemporary cities. Among these, the sustainable planning approach has gained prominence, emphasising the necessity to integrate environmental, social and economic aspects in urban development. This approach aims not only to meet the current needs of the population but also to ensure that future generations will be able to enjoy a healthy and functional urban environment ( Guillen et al., 2017; Rafieian & Kianfar, 2023; Schultheiss et al., 2024).

Furthermore, participatory planning has emerged as a significant trend, advocating for the incorporation of diverse perspectives in the decision-making process, thereby enhancing the legitimacy and efficacy of urban policies ( Hamdouch & Galvan, 2019). These developments reflect a shift towards a more holistic and collaborative model of urban planning, which contrasts with more traditional, hierarchical approaches.

A further pertinent area of study is resilience-based planning, which concentrates on the ability of urban areas to adapt to and recuperate from crises and disasters, whether of a natural or human-induced origin. This approach has become increasingly crucial in the context of climate change and rapid urbanisation, where cities must be able to cope with unpredictable challenges ( Shahsavar et al., 2022). Furthermore, the literature emphasises the significance of technology and innovation in urban planning, with an increasing focus on the utilisation of digital tools and big data to enhance urban management and citizen participation ( Wang et al., 2023). These doctrinal streams not only enrich the academic discourse, but also provide practical frameworks for the implementation of effective policies to address the complexities of today’s urban environment.

In the scientific literature, Bastos et al. (2022) present a systematic review of studies on the infrastructure of smart cities with the aim of developing citizen participation in the management and governance of cities. The review of 76 studies reveals a growing interest in developing applications to promote citizen participation in identifying urban problems and contributing to decision-making. These applications enable citizens to report on urban problems and participate in decision-making processes related to urban issues.

Samavati et al. (2024) set out to identify the factors that contribute to citizens’ happiness in urban public spaces. Their analysis of 57 articles identified 64 factors in eight domains: physical, ecological, visual, functional, subjective, political and personal. This provides a comprehensive overview of the factors influencing urban happiness. It enables policy-makers and urban planners to make informed decisions to improve the quality of life and happiness of citizens.

In their systematic review of 55 papers, Lim et al. (2019) found evidence of several studies examining the political and technological strategies employed in smart cities to enhance citizen participation, safeguard the environment, facilitate social development and promote sustainable development. These strategies have been shown to foster an increase in social capital.

While Wang et al. (2023) posit that in order to meet the needs of citizens and contribute to improvements in urban planning and construction, it is necessary to address the deficiencies of public services through the implementation of urban planning strategies that enhance the quality of life.

The problem is justified based on a review of the literature of the last decade on the effectiveness of urban planning implemented by local governments on citizen satisfaction, as it directly affects the quality of life of citizens, economic development and environmental sustainability.

As cities and their needs evolve over time, it is important to evaluate the effectiveness of urban planning so that local governments can adapt and continuously improve, adjusting strategies according to results and new realities. Effective urban planning can help reduce spatial and social inequalities and ensure that all citizens, regardless of their location or socio-economic status, have access to essential opportunities and services. The contribution of the topic under study is considered from the knowledge gap, as the effectiveness of urban planning is so far unknown. This is due to the fact that there are many limitations in local governments and their management capacity is insufficient, which has not allowed them to achieve the effectiveness of what has been designed in urban planning.

According to the reality addressed, the research questions would be the following: what has been the effectiveness of urban planning implemented by local governments on citizen satisfaction, according to the scientific literature?; what is the state of the art of the relationship between urban planning implemented by local governments and citizen satisfaction?; what is the relationship between urban planning and citizen welfare?; are there convergences in the empirical findings of researchers; and are there convergences in the empirical findings of researchers?.

The objectives of the research are: to know the effectiveness of urban planning implemented by local governments on citizen satisfaction, according to the scientific literature; to identify the state of the art of the relationship between urban planning implemented by local governments and citizen satisfaction; to know the relationship between urban planning and citizen well-being, and to describe the convergences in the empirical findings of the researchers.

## Methods

The methodology was approached from the method of systematic literature review of the phenomenon under study and the same, which allows to systematise the knowledge on the subject addressed, guiding the process of analysis and synthesis to summarise this evidence from primary sources, taking into account an established search protocol ( Page et al., 2021).

The purpose that guided the review was to know the effectiveness of urban planning implemented by local governments in terms of citizen satisfaction, as it directly affects the quality of life of citizens, economic development and environmental sustainability.

### Database search

In order to systematically answer these questions, the PRISMA (Preferred Reporting Items for Systematic reviews and Meta-Analyses) model was used ( Page et al., 2021), which deduces the procedures and protocols applied to the studies referred to the analysis under study, including conceptual and methodological aspects.

Thus, the eligibility criteria were the review of the scientific literature from three databases Scopus, Web of Science (WOS) and Dimensions, detailing the search protocol and ensuring the relevance and representativeness of scientific articles, books, reviews, conference papers, notes and book chapters; as well as the structure of the articles, which had to have the IMRD structure (Introduction, Method, Results and Discussion and Conclusions) and include the study variables in the accredited database in the academic institutions. The search strategies were not limited by time periods, and the Boléan operators for the search of information were those shown in Table 1.

**Table 1.** Databases for the systematic review of literature.

Database	Search protocol	Documents
Scopus	TITLE-ABS-KEY (urban AND planning OR urban AND order) AND TITLE-ABS-KEY (citizen AND satisfaction)	58
WOS	WOS: (“economy” AND “crime” AND “Covid-19”)	11
Dimensions	(“urban AND planning” OR “urban AND order”) AND (“Local governments”) AND (citizen AND satisfaction)	165
Total documents		234

Note. Retrieved from systematic review of databases.

Thirty-three relevant studies were selected, including scientific articles, books, reviews, conference papers, notes and book chapters. The information matrix was used for the selection of information, which included the following fields: author, title, DOI year, link, abstract, language, source, etc. The data selection process covers globally the scientific evolution on the effectiveness of urban planning implemented by local governments on citizen satisfaction, without time limits, which addressed 22 research studies accessible in full text. The inclusion criteria focused on studies that considered this relationship as their main focus, excluding those that addressed it as an afterthought or included traditional planning.

To ensure quality, the statement of the research objectives, the design of the study, the presentation of relevant data and cases, the resolution of the research questions and the appropriate discussion of the results are assessed. The main data sources are Scopus, Web of Science and Dimensions, with specific search terms: “urban planning” and “citizen satisfaction”.

For the data extraction process, the main data sources used were Scopus, Web of Science and open access Dimensions, framed in the systematic literature review and using the Excel format as a resource for the classification of the data. The duplicated, relevant, accessible and selected articles, books, reviews, conference papers, notes and book chapters were identified for the assembly of the Prisma 2020 item selection flowchart, which consists of identification, screening and inclusion ( Haddaway et al., 2022).

The criteria followed to retrieve the data for the selection of the studies were identified using the Boléan operators detailed in Table 1, based on the fact that the objective is to show the effectiveness of urban planning implemented by local governments on citizen satisfaction using the systematic literature review, which García (2015) considers to be a methodical and exhaustive approach to collect, analyse and synthesise existing research on a given topic following a structured process with the aim of minimising bias and producing more reliable results.

**Figure 1.**
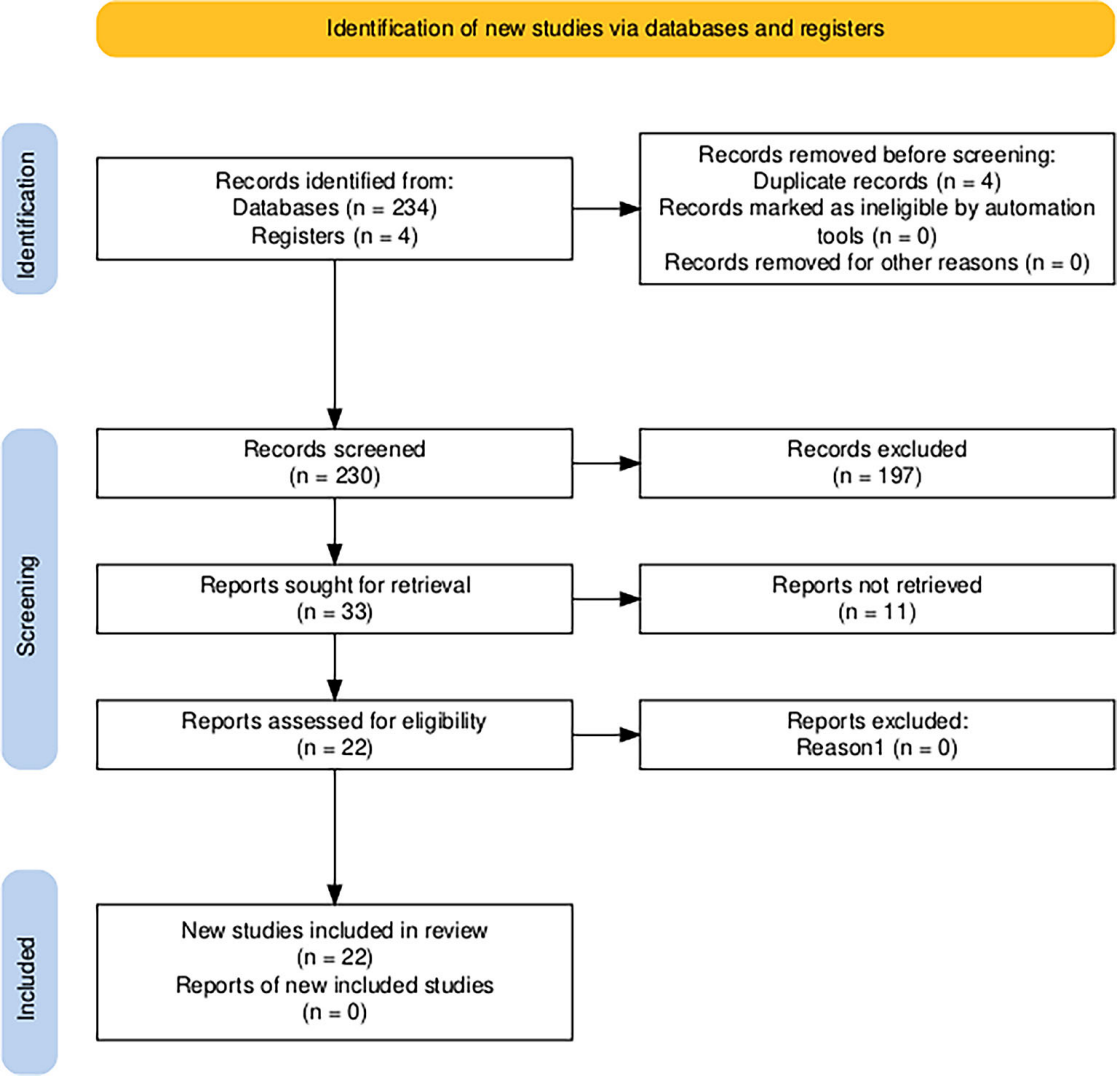
Diagram of the Prisma 2020 Flow Chart for item selection. *Note.* Article selection process for the systematic literature review using the PRISMA 2020 scheme, Page et al. (2021).

The PRISMA flowchart for this systematic review was derived from the R package and a Shiny application for generating PRISMA 2020-compliant flowcharts, which features interactivity for optimal digital transparency and open synthesis Campbell Systematic Reviews ( Page et al., 2021). It can be seen in Figure 1.

For the analysis of the risk assessment, use is made of the figure of traffic lights, taking a critical view that contributes to the research, and the tools to be used are Bibliometrix and VOSviewer.

Bibliometrix is a tool developed in R, supported by the R Core Team and the R Foundation for Statistical Computing (Bibliometrix, 2023) and requires the installation of R and Rstudio for the analysis of scientific literature to measure the development of the topic under study.

VOSviewer, developed by Leiden University, is an open source software for creating and visualising bibliometric networks. It offers text mining functionality to build co-occurrence networks of key terms ( VOSViewer, 2023).

In order to have traceability of the complete research process, complete information can be accessed at the following link zenodo: https://doi.org/10.5281/zenodo.13904617 ( Llonto et al., 2024).

## Results

A theoretical and epistemological contribution was made to the topic under study, for which bibliometrics was used as part of the heuristics of the state of the art, considering the contribution of hermeneutics in the immersion into the content of the related literature ( Báez, 2023; Pérez, 2023).

**Figure 2.**
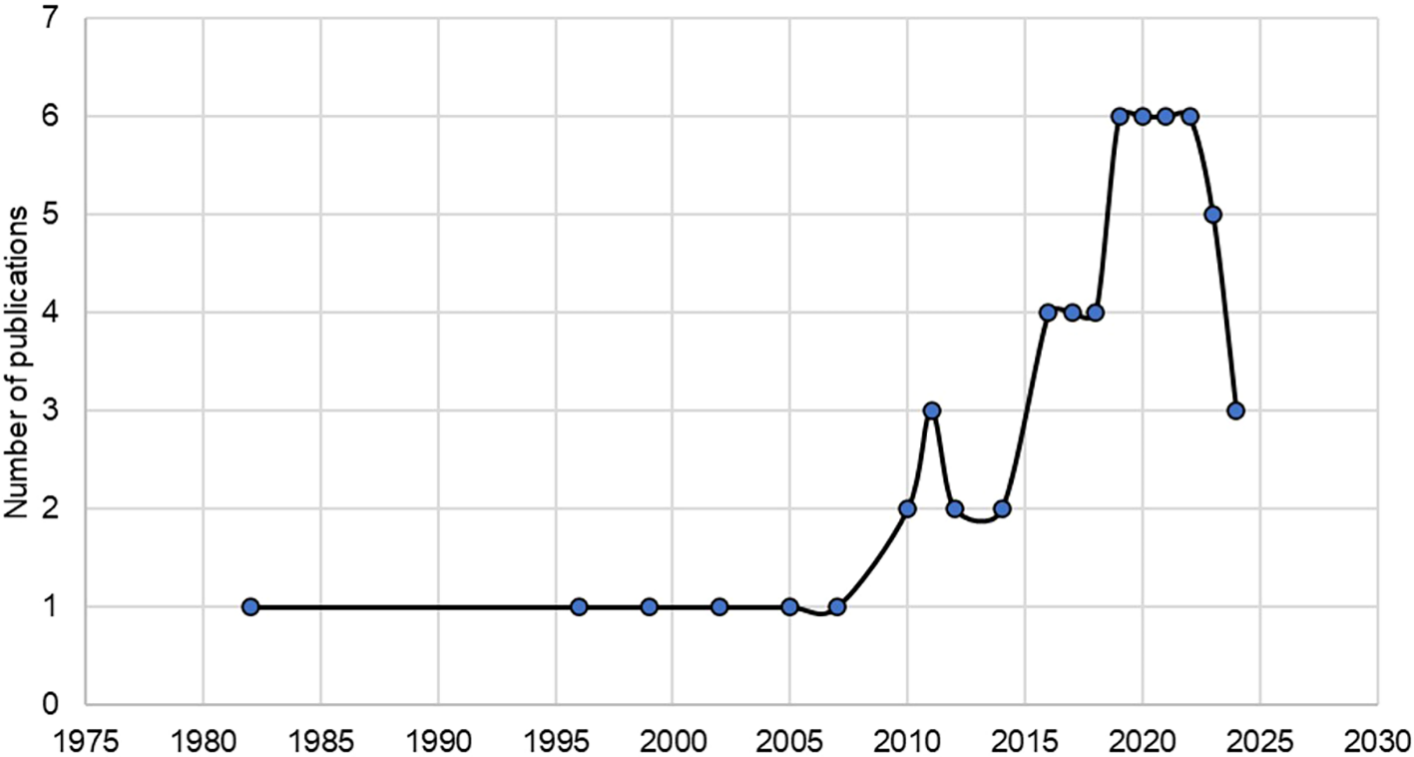
Developments in publications on urban planning and citizen welfare. *Note.* Retrieved from Scopus database.

The relationship between urban planning and citizen well-being started in 1982, with increasing interest until 2024. Such an increase has generated a trend in revealing the effectiveness of urban planning implemented by local governments on citizen satisfaction over the last decade for social demands increasingly noticeable as of 2019 ( Figure 2).

**Figure 3.**
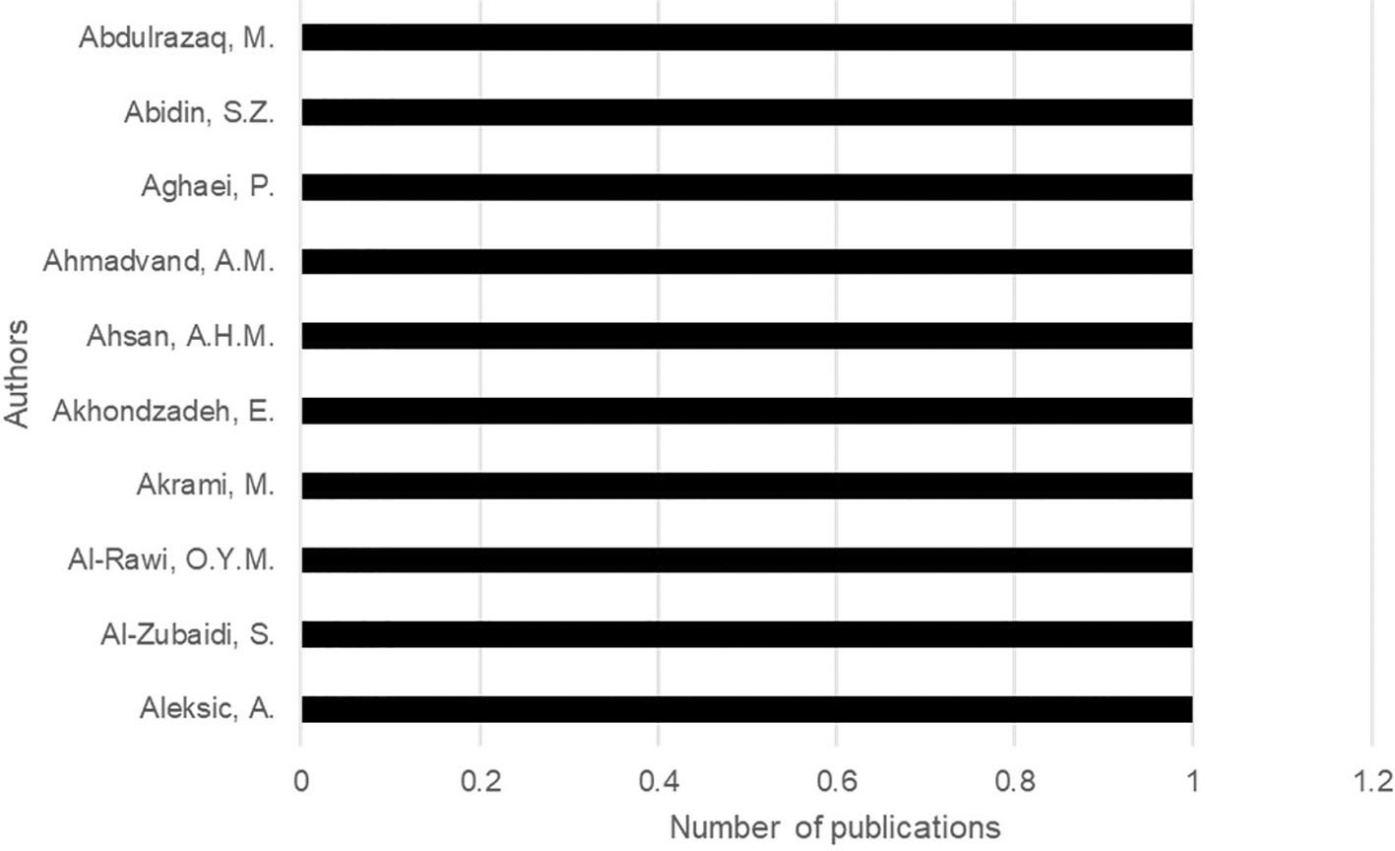
Main authors contributing to the collection. *Note.* Retrieved from Scopus database.

The most relevant authors are Abdulrazaq from Baghdad University in Baghdad, Abidin from Teknologi Mara University in Malaysia, Aghaei from Isfahan University of Technology in Australia ( Figure 3).

**Figure 4.**
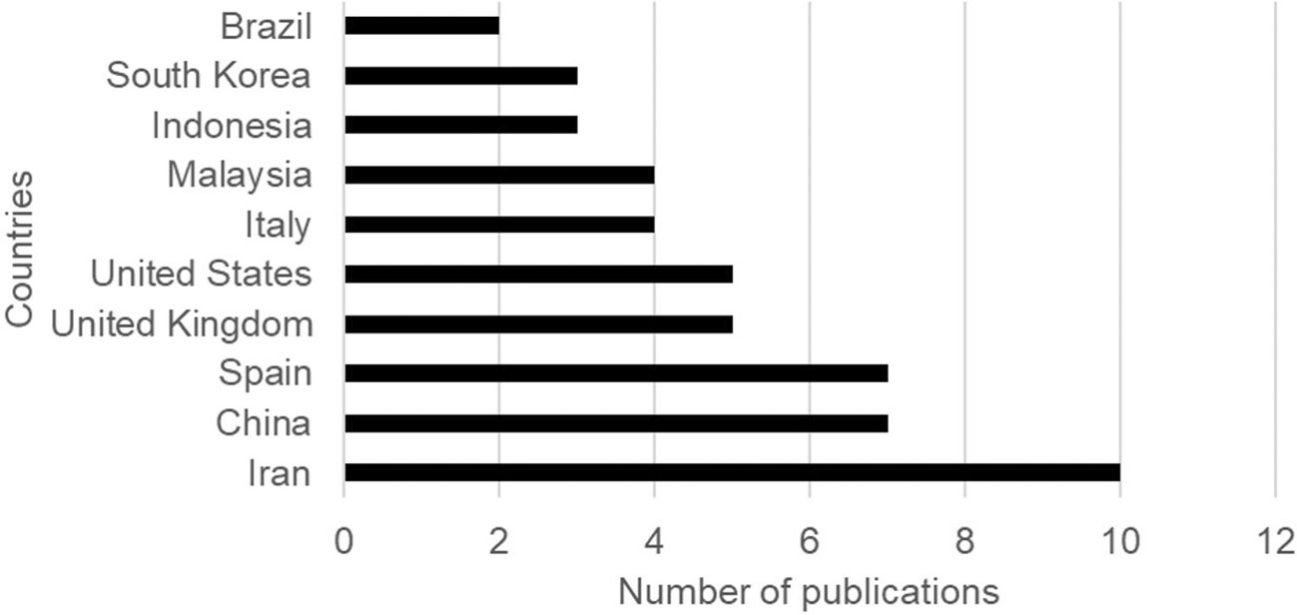
Publications by country on urban planning and citizen well-being. *Note.* Retrieved from Scopus database.

In the analysis of publications by country, the largest contributions to the literature are concentrated in the countries of Iran (13%), China (9%), Spain (9%), the United Kingdom (6%), the United States (6%) and Italy (5%) on the relationship between urban planning and citizen well-being ( Figure 4).

For the analysis of the authors’ contribution we use Lotka’s Law, a mathematical regularity proposed by Alfred J. Lotka in 1926, which describes the frequency of publication in a scientific field. Considering this law, a small group of authors with the highest production generates most of the scientific literature in a given area, being 100% who produce an article.

Bradford’s law, also called the law of dispersion of scientific literature, proposed by Samuel C. Bradford in 1934, describes the distribution of articles on a topic in scientific journals. It highlights the existence of a core group of journals that concentrate most of the relevant articles on a specific topic, which makes them the main sources of expertise ( Table 2).

**Table 2.** Bradford Law.

Magazine	Ranking	Frequency	Cumulative frequency	Zone
INTERNATIONAL JOURNAL OF HUMAN CAPITAL IN URBAN MANAGEMENT	1	2	2	Zone 1
IOP CONFERENCE SERIES: EARTH AND ENVIRONMENTAL SCIENCE	2	2	4	Zone 1
JOURNAL OF ENVIRONMENTAL ENGINEERING AND LANDSCAPE MANAGEMENT	3	2	6	Zone 1
PLOS ONE	4	2	8	Zone 1
17TH ITS WORLD CONGRESS	5	1	9	Zone 1
2020 INTERNATIONAL WIRELESS COMMUNICATIONS AND MOBILE COMPUTING, IWCMC 2020	6	1	10	Zone 1
2022 IEEE INTERNATIONAL CONFERENCE ON AUTOMATIC CONTROL AND INTELLIGENT SYSTEMS, I2CACIS 2022 - PROCEEDINGS	7	1	11	Zone 1
2024 47TH ICT AND ELECTRONICS CONVENTION, MIPRO 2024 - PROCEEDINGS	8	1	12	Zone 1
ADVANCES IN SCIENCE, TECHNOLOGY AND INNOVATION	9	1	13	Zone 1
AIP CONFERENCE PROCEEDINGS	10	1	14	Zone 1

Note: Obtaining the Scopus database from Bibliometrix.

**Figure 5.**
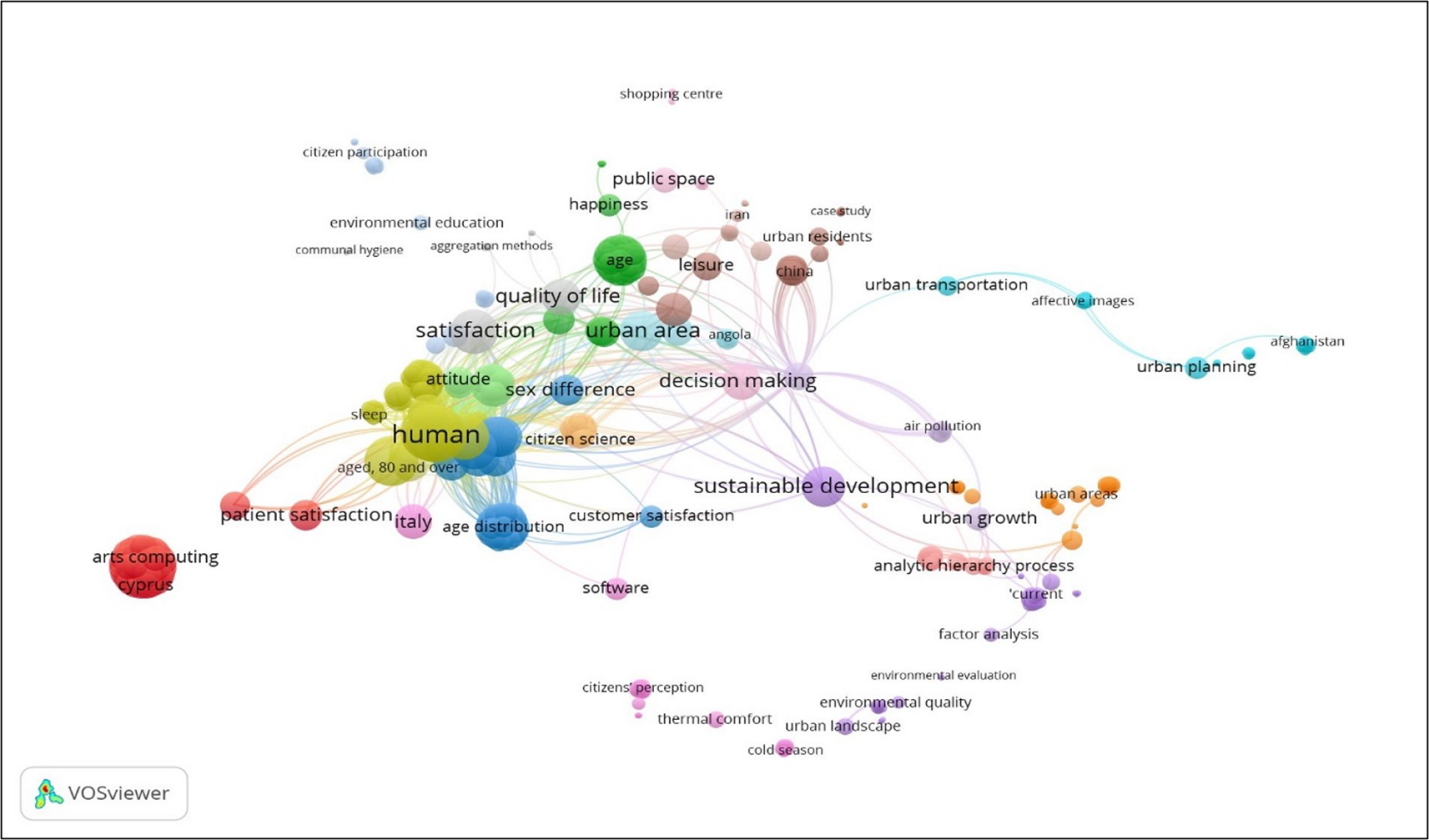
Semantic map of the relationship between urban planning implemented by local governments and citizen satisfaction. *Note.* Obtained from the open source programme VOSviewer, with metadata from Scopus.

For the state of the art of the relationship between urban planning implemented by local governments and citizen satisfaction, a semantic analysis is presented based on the key terms used in the research and the relationships between their authors, journals, sponsors, institutional affiliations and other metadata, which in an underlying way generate a dynamic in a research ecosystem and, In the analysis it is evident that both variables have a close relationship, where at the same time the clusters indicate a closeness between variables such as sustainable development, happiness, public space, urban growth, urban areas, satisfied customers, urban residents, among others. The construction of semantic maps uses the arguments proposed by Zipf’s law, also known as the law of least effort, which is an empirical law proposed by the American linguist George Kingsley Zipf in 1949. This law refers to the frequency of occurrence of words in a text or linguistic corpus ( Figure 5).

The contribution of the relationship between urban planning implemented by local governments and citizen satisfaction is agreed to have a positive effect when considering a planned city that generates effective conditions for improved development outcomes ( Liu et al., 2022; Orr & West, 2002; Sánchez et al., 2021).

The systematic literature review has identified three main building blocks in the contribution of urban planning to citizen satisfaction. These blocks highlight the importance of planning effectiveness in providing a solid basis for future research into the design of local public policies, as shown in Table 3.

**Table 3.** Outcome of the individual studies.

Authors	Contribution block	Urban planning guidelines
Aleksic et al. (2019); Colderley (1999); Croese, S. and Pitcher, M (2019); Fang et al. (2020)	**Urban planning as a catalyst for the impact of citizen satisfaction.** Planning has been a relevant contribution to local policy formulation considering city master plans and use plans; being crucial for quality of life; having a new approach to Neoliberalism.	Development of local policies based on the neoliberal approach to smart urban development.
Jackson (2018); Lo Piccolo (2017); Niu et al. (2017); Orr, M and West, D (2002); Sánchez et al. (2021); Santos et al. (2007)	**Theoretical input for urban planning.** Theoretical input is based on Neoliberalism for urban and regional planning; as well as modernist planning and a new multidimensional construct with a multidimensional nature for urban revitalisation considering synthetic indicators for quality of life and considering a complementary approach.	The various theoretical contributions contextualise a modernist planning based on a multidimensional aspect to address quality of life for modern well-being.
Silva (2020); Silva (2016); Stankovic et al. (2017); Ulloa (2016); Varró, K and Szalai A (2022); Wang et al.(2023); Wang, Z and Zhong, Y. (2020); Yermachenko, V. (2023); Xue, J and Kębłowski, W (2022).	**Smart planning for citizen satisfaction.** Planning tools and effective transformation of the territory based on smart planning enable the development of a smart city for spatial planning system in co-constitutive performance for centralised urban development **.** Emphasising smart governance for socio-spatial development, considering a socio-ecological transformation to contribute to optimal urbanism and well-being of life.	Deepen the design and implementation of public governance reforms aimed at optimising the management of urban planning at the local level for smart urban city development.

Note: Obtaining the analysed database.

The first block entitled “Urban planning as a catalyst for the impact of citizen satisfaction”, studies agree that planning is a relevant input for the formulation of local policies to improve the quality of life of citizens with a neoliberal approach ( Aleksic et al., 2019; Colderley, 1999; Croese & Pitcher, 2019; Fang et al., 2020).

In the second block “Theoretical contributions for urban planning” reveals that the relevant contributions of the authors planning focuses on Neoliberalism with a multidimensional construct that reveals to have key indicators for multidimensional development that contributes to modern well-being and considers the well-being of citizens to deepen the design of policies ( Jackson, 2018; Lo Piccolo, 2017; Niu et al., 2017; Orr & West, 2002; Sánchez et al., 2021; Santos et al., 2007).

The third block “Smart planning for citizen satisfaction” involves the authors’ consensus that planning tools serve for the effective transformation of the territory considering smart planning for the development of a spatial planning system where smart governance stands out, which is transformed into optimal urbanism and a timely and development-generating socio-spatial development.

In this context, the effectiveness of urban planning implemented by local governments on citizen satisfaction is still deficient, given that in the multilevel and smart governance environment a multidimensional approach is required where the contribution to quality of life allows inferring the new modernist planning approach to achieve the development of cities in line with improved policy making and leads to the establishment of new and timely approaches to urban planning based on new public governance.

## Discussion

The contribution of the research reveals the synthesis and systematisation of the state of the art evidence on the positive relationship of local government urban planning on citizen satisfaction. A detailed review of the literature has identified significant convergences in the empirical findings of various researchers in different geographical contexts. This exhaustive literature review has allowed us to identify convergence in the empirical findings that originate from different geographical contexts. This review is grouped into contributions from three main thematic blocks, which involves facilitating the understanding of the diverse theoretical approach linked to sustainable development, happiness, public space, urban growth, urban areas, satisfied customers and urban residents, generating greater effectiveness in the territorial environment.

The contribution of the positive and substantive relationship between urban planning and citizen satisfaction, the systematic review of the literature reveals a widespread consensus among researchers, which gives rise to the importance of urban planning. In line with this result, participatory planning has emerged as a key trend, promoting the inclusion of diverse voices in the decision-making process, which translates into greater legitimacy and effectiveness of urban policies ( Hamdouch & Galvan, 2019). Likewise Shahsavar et al. (2022) engages resilience-based planning, which focuses on the capacity of cities to adapt to and recover from crises and disasters, whether natural or human-induced. This approach has become increasingly crucial in a context of climate change and rapid urbanisation, where cities must be able to cope with unpredictable challenges. The literature also highlights the importance of technology and innovation in urban planning, with a growing interest in the use of digital tools and big data to improve urban management and citizen participation ( Wang et al., 2023).

The main limitation of systematic literature reviews is the possible omission of relevant studies not indexed in the databases used. Although the Scopus, WOS and Dimensions databases with wide coverage were used, future reviews could consider incorporating other sources. Since it is important that this is a multidimensional phenomenon, the keyword search may miss some studies that use different terminology.

In terms of future research perspectives, it is recommended to deepen empirical studies, possibly quantitative, that examine the relative weight of the different dimensions of urban planning that contribute to citizen satisfaction and assess the effectiveness of urban planning in the territories.

## Conclusions

The analysis of the systematic literature review involves a current analysis of research on urban planning and citizen satisfaction, being a relatively new field, publications were born in the 1980s with a growing interest from 2019 onwards that increased significantly with a sustained increase. The most prominent authors are Abdulrazaq of Baghdad, Abidin from Malaysia and Aghaei from Australia, who address issues of form from various perspectives in the global arena.

Compliance with Lotka, Bradford and Zipf’s bibliometric laws is evidence of the solidity and maturity that this line of research is acquiring. Likewise, semantic analysis by means of conceptual maps identifies closely linked thematic clusters, such as sustainable development, happiness, public space, urban growth, urban areas, satisfied customers, urban residents, among others, which makes evident the theoretical-conceptual relationship between the effectiveness of urban planning and citizen satisfaction.

The contribution of the substantive relationship between urban planning and citizen satisfaction, the systematic review of the literature reveals a general consensus among researchers. There is strong empirical support that modern planning is understood as effectiveness that is characterised in a territory involving sustainable development under a smart cities approach that requires a different look at the theoretical-conceptual input into planning development.

The systematic literature review has identified three main building blocks in the contribution of urban planning to citizen satisfaction. These blocks highlight the importance of planning effectiveness to provide a solid basis for future research.

The findings in the various empirical contributions find converging in three main blocks the contributions related to: (i) Urban planning as a catalyst for the impact of citizen satisfaction, given that using the tool of planning will lead to the development of local policies based on the Neoliberalism approach for smart urban development; (ii) Theoretical contributions for urban planning that contextualises a modernist planning based on a multidimensional aspect to address quality of life for modern well-being and (iii) Smart planning for citizen satisfaction for the design and implementation of public governance reforms aimed at optimising urban planning management at the local level for smart urban city development.

It is concluded that the scientific evidence addressed confirms the thesis that planning has been used in a useful and effective way in various local governments under the paradigm of a modernist planning considering the multidimensional aspect for the development of an intelligent planning that channels a multilevel governance for sustainable development impact oriented towards the objectives of economic and social development, This systematic review lays a solid foundation for future research and the design of local public policies aimed at strengthening governance in order to optimise the use of public resources.

### Ethics and consent

Ethics and consent were not required for the performed study.

## Data Availability

Zenodo: Urban planning effectiveness and citizen satisfaction. A systematic literature review. Version 4. https://doi.org/10.5281/zenodo.13901189 ( Llonto et al., 2024). The project contains the following underlying data:
• Author Contributions.xlsx (Results of the analysis of the contributions by author and by blocks). • Bibliometric figures.xlsx (Results of the data in tables and figures obtained from the databases). • Dimensions Data.xlsx (Raw data from the Dimensions database). • General data of the RSL process.xlsx (Results of the systematic literature review from Scopus, Web Of Science, Dimensions database). • ResEB.xlsx (Matrix of development of scientific information search equations). • Scopus database analysis.xlsx (Result of the search equation by affiliation, year, country, subject area, type of document and author). • Scopus database.xlsx (Processed data from the Scopus database). • Documents from the Data RSL on Urban Planning and Satisfaction (Final list of selected documents). Zenodo: Urban planning effectiveness and citizen satisfaction. A systematic literature review. Version 4. https://doi.org/10.5281/zenodo.13904617 ( Llonto et al., 2024). This project contains the following extended data:
• Supplementary Figure 1. (Diagram of the Prisma 2020 Flow Chart for item selection). • Supplementary Figure 2. (Developments in publications on urban planning and citizen welfare). • Supplementary Figure 3. (Main authors contributing to the collection) • Supplementary Figure 4. (Publications by country on urban planning and citizen well-being). • Supplementary Figure 5: (Semantic map of the relationship between urban planning implemented by local governments and citizen satisfaction). Zenodo: PRISMA checklist for ‘Urban planning effectiveness and citizen satisfaction. A systematic literature review’. Version 4. https://doi.org/10.5281/zenodo.13904617 ( Llonto et al., 2024). Data are available under the terms of the Creative Commons Zero “No rights reserved” data waiver (CC0 1.0 Public domain dedication).
