# Urban planning effectiveness and citizen satisfaction. A systematic literature review

**DOI:** 10.12688/f1000research.157550.2

**Published:** 2025-10-16

**Authors:** Yefferson Llonto Caicedo, Rogger Orlando Morán Santamaría, Guido Alarcón Villanueva, Leticia Noemi Zavaleta Gonzáles, Willy Darwin Llatas Díaz, Ida Blanca Pacheco Gonzales, Rocío Janet Pejerrey González, Percy Junior Castro Mejía, Carlos William Atalaya Urrutia

**Affiliations:** 1 Lambayeque, Universidad Nacional Pedro Ruiz Gallo, Lambayeque, Lambayeque, Peru; 2 La Libertad, Universidad Cesar Vallejo, Trujillo, La Libertad, Peru; 3 Cajamarca, Universidad Nacional de Cajamarca, Cajamarca, Cajamarca, Peru; 4 Lambayeque, Universidad Senor de Sipan, Chiclayo, Lambayeque, Peru

**Keywords:** PRISMA, bibliometrics, bibliometrix, urban planning, citizen satisfaction, systematic review, state of the art.

## Abstract

**Background:**

The population is increasingly demanding a better quality of life in their territories, which requires better urban planning. This study aims to find out the effectiveness of urban planning implemented by local governments on citizen satisfaction.

**Method:**

A systematic literature review was conducted exploring the evolution of the state-of-the-art databases in Scopus, WOS and Dimensions, involving a relevant selection of empirical studies on the effectiveness of urban planning on citizen satisfaction, using quality criteria and the application of the PRISMA diagram.

**Results:**

The findings in the various empirical contributions find converging in three main blocks the contributions related to: (i) Urban planning as a catalyst for the impact of citizen satisfaction, given that using the planning tool will lead to the development of local policies based on the Neoliberalism approach for smart urban development; (ii) Theoretical contributions for urban planning that contextualises a modernist planning based on a multidimensional aspect to address quality of life for modern well-being and (iii) Smart planning for citizen satisfaction for the design and implementation of public governance reforms aimed at optimising urban planning management at the local level for smart urban city development.

**Conclusion:**

There is strong theoretical and empirical support that is closely linked to sustainable development, happiness, public space, urban growth, urban areas, satisfied customers and urban residents. Future research should examine the relative weight of urban planning dimensions and effectiveness in the sustainable development of territories and citizen satisfaction.

## Introduction

In recent decades, urban planning has acquired a heightened level of relevance due to the phenomenon of urbanisation and the associated challenges that it presents, including those pertaining to environmental sustainability, urban mobility and the quality of life of citizens ( Bhuiyan & Islam, 2023; Sanchez et al., 2024; Canal et al., 2023). The effectiveness of urban planning has become a crucial issue to ensure more liveable and resilient cities. Globally, efforts have been directed towards integrating advanced technologies and innovative concepts, such as smart cities, which aim to optimise the management of urban resources and improve resident satisfaction ( Supangkat et al., 2023; Papageorgiou et al., 2024; Alfaro-Navarro et al., 2024). Nevertheless, the implementation of these plans does not always result in the improvements that citizens perceive, which gives rise to questions about the relationship between urban planning and citizen satisfaction ( Lim et al., 2019).

In particular, citizen satisfaction has emerged as a pivotal metric for evaluating the efficacy of urban interventions ( Wang et al., 2023). A number of studies have examined the impact of infrastructure, public services and sustainability initiatives on the perception and well-being of inhabitants. However, the results are variable and frequently contingent on the specific context of each city. This lack of consensus in the literature reflects the complexity of urban dynamics and the necessity for more holistic and personalised approaches that consider the expectations and needs of communities.

Furthermore, research has demonstrated that the incorporation of green spaces and recreational areas into urban planning strategies has a considerable positive effect on citizen satisfaction. Such spaces not only enhance the visual appeal of urban environments but also confer tangible benefits for the mental and physical well-being of residents ( Rico et al., 2022).

The quality of infrastructure and the availability of public services, such as transport, education and health, are pivotal factors in determining levels of citizen satisfaction. The implementation of enhanced transport infrastructure has been demonstrated to diminish travel times and, moreover, to enhance the general perception of governmental efficiency.

Urban planning represents a crucial instrument for local governments in their pursuit of enhancing the quality of life of their citizens. The integration of elements of sustainability, participation and infrastructure development has been demonstrated to be the most effective strategy for increasing citizen satisfaction. It is recommended that future studies adopt an interdisciplinary approach combining quantitative and qualitative data in order to gain a deeper understanding of the factors influencing urban satisfaction.

Notwithstanding the endeavours to enhance the quality of urban life, there remain deficiencies in our comprehension of the manner in which diverse urban planning strategies impact citizen satisfaction. Recent studies have indicated that while certain urban planning strategies may improve specific aspects of urban life, such as mobility or access to services, there is not always a direct correlation with increased overall resident satisfaction. This is due to the fact that the effectiveness of such planning strategies is currently unknown, given the numerous constraints faced by local governments and the weak management capacities that often result in ineffectiveness ( Guillen et al., 2017; Supangkat et al., 2023).

The objective of this study is to conduct a comprehensive review of the existing literature on the effectiveness of urban planning implemented by local governments in influencing citizen satisfaction. This is a crucial issue as citizen satisfaction directly impacts the quality of life of citizens, economic development and environmental sustainability.

The theoretical contribution of the currents suggests that the effectiveness of urban planning is influenced by a number of theoretical and doctrinal factors, including the adoption of participatory approaches, the integration of smart technologies and the incorporation of social equity considerations in decision-making processes ( Sohail et al., 2005; Wu et al., 2022).

The field of urban planning has undergone significant evolution over the past few decades, giving rise to a multitude of doctrinal approaches that seek to address the complex challenges facing contemporary cities. Among these, the sustainable planning approach has gained prominence, emphasising the necessity to integrate environmental, social and economic aspects in urban development. This approach aims not only to meet the current needs of the population but also to ensure that future generations will be able to enjoy a healthy and functional urban environment ( Guillen et al., 2017; Rafieian & Kianfar, 2023; Schultheiss et al., 2024).

Furthermore, participatory planning has emerged as a significant trend, advocating for the incorporation of diverse perspectives in the decision-making process, thereby enhancing the legitimacy and efficacy of urban policies ( Hamdouch & Galvan, 2019). These developments reflect a shift towards a more holistic and collaborative model of urban planning, which contrasts with more traditional, hierarchical approaches.

A further pertinent area of study is resilience-based planning, which concentrates on the ability of urban areas to adapt to and recuperate from crises and disasters, whether of a natural or human-induced origin. This approach has become increasingly crucial in the context of climate change and rapid urbanisation, where cities must be able to cope with unpredictable challenges ( Shahsavar et al., 2022). Furthermore, the literature emphasises the significance of technology and innovation in urban planning, with an increasing focus on the utilisation of digital tools and big data to enhance urban management and citizen participation ( Wang et al., 2023). These doctrinal streams not only enrich the academic discourse, but also provide practical frameworks for the implementation of effective policies to address the complexities of today’s urban environment.

In the scientific literature, Bastos et al. (2022) present a systematic review of studies on the infrastructure of smart cities with the aim of developing citizen participation in the management and governance of cities. The review of 76 studies reveals a growing interest in developing applications to promote citizen participation in identifying urban problems and contributing to decision-making. These applications enable citizens to report on urban problems and participate in decision-making processes related to urban issues.

Samavati et al. (2024) set out to identify the factors that contribute to citizens’ happiness in urban public spaces. Their analysis of 57 articles identified 64 factors in eight domains: physical, ecological, visual, functional, subjective, political and personal. This provides a comprehensive overview of the factors influencing urban happiness. It enables policy-makers and urban planners to make informed decisions to improve the quality of life and happiness of citizens.

In their systematic review of 55 papers, Lim et al. (2019) found evidence of several studies examining the political and technological strategies employed in smart cities to enhance citizen participation, safeguard the environment, facilitate social development and promote sustainable development. These strategies have been shown to foster an increase in social capital.

While Wang et al. (2023) posit that in order to meet the needs of citizens and contribute to improvements in urban planning and construction, it is necessary to address the deficiencies of public services through the implementation of urban planning strategies that enhance the quality of life.

The problem is justified based on a review of the literature of the last decade on the effectiveness of urban planning implemented by local governments on citizen satisfaction, as it directly affects the quality of life of citizens, economic development and environmental sustainability.

As cities and their needs evolve over time, it is important to evaluate the effectiveness of urban planning so that local governments can adapt and continuously improve, adjusting strategies according to results and new realities. Effective urban planning can help reduce spatial and social inequalities and ensure that all citizens, regardless of their location or socio-economic status, have access to essential opportunities and services. The contribution of the topic under study is considered from the knowledge gap, as the effectiveness of urban planning is so far unknown. This is due to the fact that there are many limitations in local governments and their management capacity is insufficient, which has not allowed them to achieve the effectiveness of what has been designed in urban planning.

According to the reality addressed, the research questions would be the following: what has been the effectiveness of urban planning implemented by local governments on citizen satisfaction, according to the scientific literature?; what is the state of the art of the relationship between urban planning implemented by local governments and citizen satisfaction?; what is the relationship between urban planning and citizen welfare?; are there convergences in the empirical findings of researchers; and are there convergences in the empirical findings of researchers?.

The objectives of the research are: to know the effectiveness of urban planning implemented by local governments on citizen satisfaction, according to the scientific literature; to identify the state of the art of the relationship between urban planning implemented by local governments and citizen satisfaction; to know the relationship between urban planning and citizen well-being, and to describe the convergences in the empirical findings of the researchers.

## Methods

The methodology was approached from the method of systematic literature review of the phenomenon under study and the same, which allows to systematise the knowledge on the subject addressed, guiding the process of analysis and synthesis to summarise this evidence from primary sources, taking into account an established search protocol ( Page et al., 2021).

The purpose that guided the review was to know the effectiveness of urban planning implemented by local governments in terms of citizen satisfaction, as it directly affects the quality of life of citizens, economic development and environmental sustainability.

### Database search

In order to systematically answer these questions, the PRISMA (Preferred Reporting Items for Systematic reviews and Meta-Analyses) model was used ( Page et al., 2021), which deduces the procedures and protocols applied to the studies referred to the analysis under study, including conceptual and methodological aspects.

Thus, the eligibility criteria were the review of the scientific literature from three databases Scopus, Web of Science (WOS) and Dimensions, detailing the search protocol and ensuring the relevance and representativeness of scientific articles, books, reviews, conference papers, notes and book chapters; as well as the structure of the articles, which had to have the IMRD structure (Introduction, Method, Results and Discussion and Conclusions) and include the study variables in the accredited database in the academic institutions. The search strategies were not limited by time periods, and the Boléan operators for the search of information were those shown in Table 1.

**Table 1.** Databases for the systematic review of literature.

Database	Search protocol	Documents
Scopus	TITLE-ABS-KEY (urban AND planning OR urban AND order) AND TITLE-ABS-KEY (citizen AND satisfaction)	58
WOS	WOS: (“urban planning” AND “spatial planning” AND “urban governance” AND “citizen satisfaction” AND “resident satisfaction”)	11
Dimensions	(“urban AND planning” OR “urban AND order”) AND (“Local governments”) AND (citizen AND satisfaction)	165
Total documents		234

Note. Retrieved from systematic review of databases.

The dataset was then divided into 234 records, as outlined in Table 1. Following a thorough review, four duplicate records were identified and eliminated, resulting in a final total of 230 records. Following the exclusion of 197 records deemed irrelevant based on their title and abstract (85.7%), 33 records were retained as potentially relevant (14.3%).

In the eligibility phase, 22 records (66.7%) had accessible full texts and proceeded to formal quality assessment, where 11 were found to be inaccessible. These counts are documented in the extraction matrix, available at the following link: General data of the RSL process.xlsx; with DOI in Zenodo: https://doi.org/10.5281/zenodo.17308186 ( Llonto et al., 2025), which allows the decision flow to be replicated.

The quality assessment was conducted using a checklist comprising six items, which were organised into two domains. The checklist items were scored on an ordinal scale (No = 0; Partial = 0.5; Yes = 1) per study. The information available in the full text and abstract was classified into two assessments.

• The thematic assessment comprises two items. The objectives of this study are twofold: (i) firstly, to examine the relationship between urban planning and citizen satisfaction; and (ii) secondly, to establish an explicit and operational link between the two variables.
• The following four items comprise the methodological evaluation: (iii) The design of the study must be consistent with the objectives of the research. (iv) The results of the study must be supported by empirical data. (v) The research questions and findings must be consistent with each other. (vi) There must be adequate discussion of the results and their limitations.

Utilising these ratings, two sub-scores are derived: thematic evaluation and methodological evaluation. The total score is then determined as the weighted sum of both domains. Prior to the implementation of the instrument, an inclusion threshold of 2.3 points in the total score was predefined. This criterion serves to reduce subjectivity and to avoid incorporating evidence from weak records. A total of 22 studies exceeded this threshold, with a score equal to 2.3, and were included in the qualitative synthesis; the rest were excluded for not meeting the minimum quality requirements.

In order to reinforce the accuracy of the procedure: The title and score were based on a standardised extraction matrix. Each item on the checklist was justified with textual evidence from the article (objectives, methods, results and discussion) recorded in the matrix. Disagreements between reviews were resolved by documented consensus.

The main data sources are Scopus, Web of Science and Dimensions, with specific search terms: “urban planning” and “citizen satisfaction”.

For the data extraction process, the main data sources used were Scopus, Web of Science and open access Dimensions, framed in the systematic literature review and using the Excel format as a resource for the classification of the data. The duplicated, relevant, accessible and selected articles, books, reviews, conference papers, notes and book chapters were identified for the assembly of the Prisma 2020 item selection flowchart, which consists of identification, screening and inclusion ( Haddaway et al., 2022).

The criteria followed to retrieve the data for the selection of the studies were identified using the Boléan operators detailed in Table 1, based on the fact that the objective is to show the effectiveness of urban planning implemented by local governments on citizen satisfaction using the systematic literature review, which García (2015) considers to be a methodical and exhaustive approach to collect, analyse and synthesise existing research on a given topic following a structured process with the aim of minimising bias and producing more reliable results.

**Figure 1.**
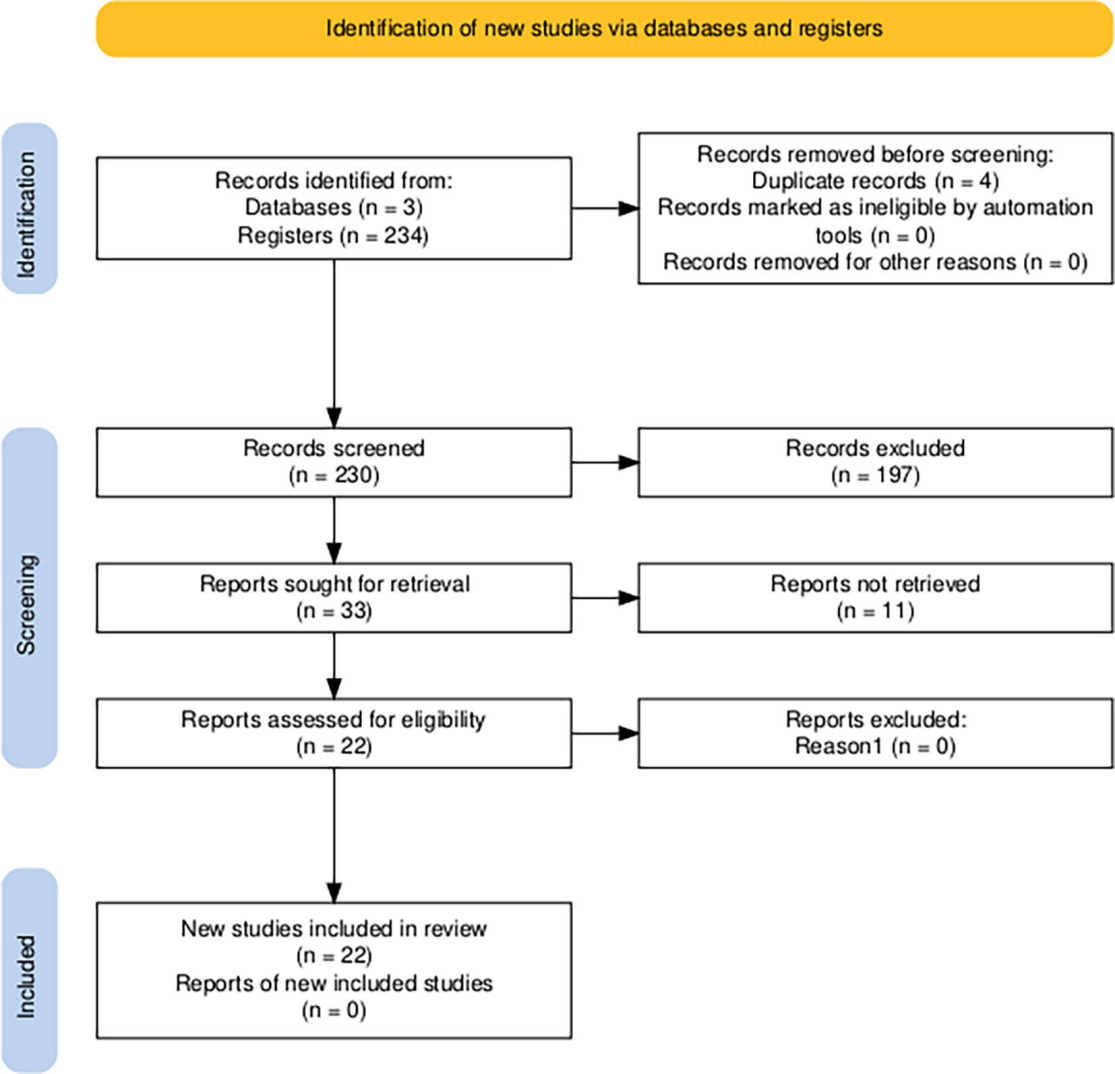
Diagram of the Prisma 2020 Flow Chart for item selection. *Note.* Article selection process for the systematic literature review using the PRISMA 2020 scheme, Page et al. (2021).

The PRISMA flowchart for this systematic review was derived from the R package and a Shiny application for generating PRISMA 2020-compliant flowcharts, which features interactivity for optimal digital transparency and open synthesis Campbell Systematic Reviews ( Page et al., 2021). It can be seen in Figure 1.

The data obtained from Scopus, Web of Science, and Dimensions was exported in CSV format and converted to Excel for processing. The analysis was conducted utilising the Bibliometrix package in R, a software that facilitates the calculation of scientific productivity metrics, including the number of documents per year and the identification of the most influential authors and institutions ( Aria & Cuccurullo, 2017). The application of VOSviewer is to visualise collaboration networks and keyword associations. This application facilitates the implementation of clustering and association strength normalisation algorithms, which are used to identify thematic clusters and co-authorship groups ( Van Eck & Waltman, 2010).

Bibliometrix is a tool developed in R, supported by the R Core Team and the R Foundation for Statistical Computing (Bibliometrix, 2023) and requires the installation of R and Rstudio for the analysis of scientific literature to measure the development of the topic under study.

VOSviewer, developed by Leiden University, is an open source software for creating and visualising bibliometric networks. It offers text mining functionality to build co-occurrence networks of key terms ( VOSViewer, 2023).

In order to have traceability of the complete research process, complete information can be accessed at the following link zenodo: https://doi.org/10.5281/zenodo.17308186 ( Llonto et al., 2025).

The present review is subject to three obvious limitations. Firstly, the heterogeneity in the operationalisation of satisfaction and in the planning instruments reduces comparability and discourages meta-analysis. This limitation is mitigated through the use of structured narrative synthesis and quality weighting. Secondly, the final size is indicative of the rigorous focus on empirical evidence and the terminological dispersion of the field. To mitigate this, we have expanded synonyms, snowballed, and established an open repository of the protocol and intermediate results for scrutiny. Thirdly, despite the utilisation of three high-coverage databases (Scopus, WoS, and Dimensions) and the standardisation of queries, the risk of publication and omission bias persists. This risk is mitigated by conducting a manual search of reference journals and ensuring full transparency of search strings and exclusion criteria. In summary, these limitations do not invalidate the findings, but rather delimit their generalisation and establish guidelines for future meta-evaluations that use more homogeneous metrics.

## Results

A theoretical and epistemological contribution was made to the topic under study, for which bibliometrics was used as part of the heuristics of the state of the art, considering the contribution of hermeneutics in the immersion into the content of the related literature ( Báez, 2023; Pérez, 2023).

**Figure 2.**
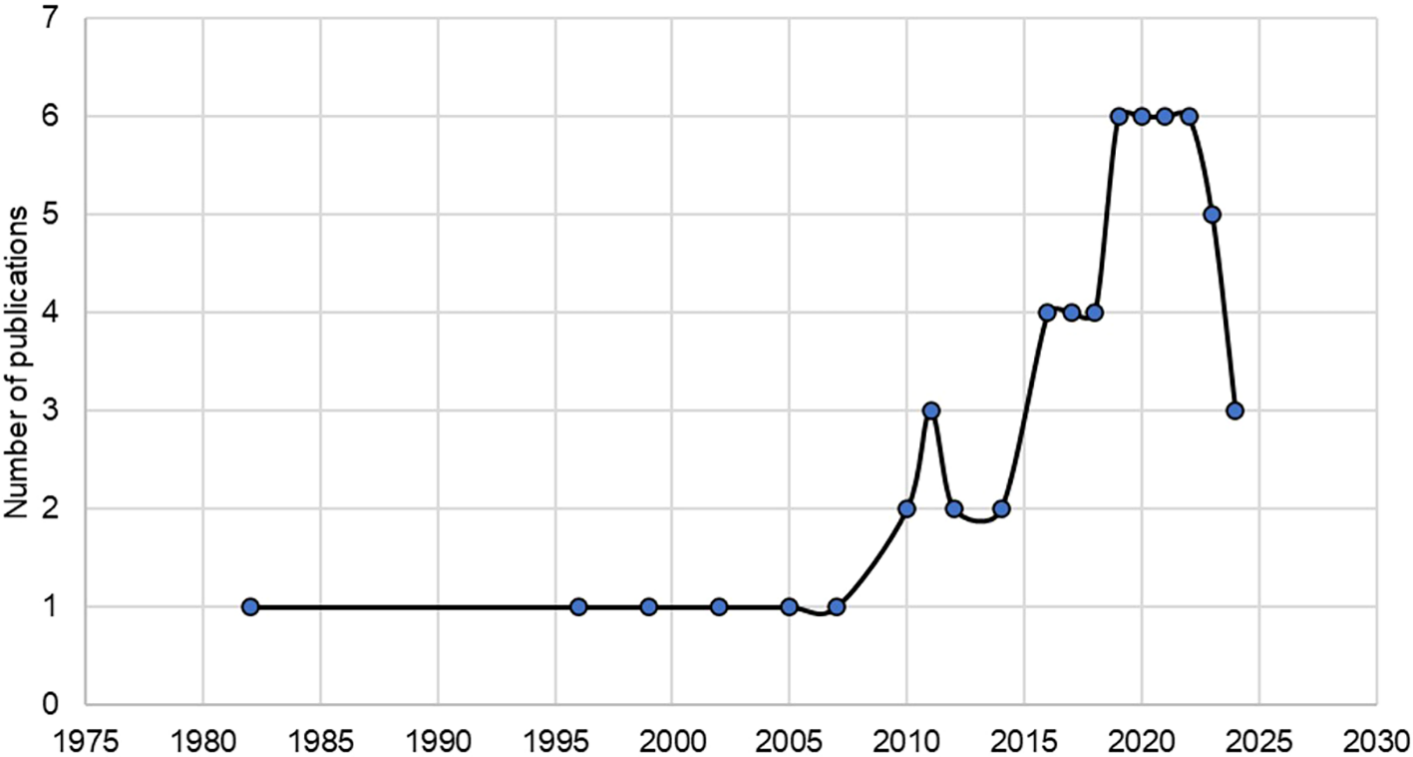
Developments in publications on urban planning and citizen welfare. *Note.* Retrieved from Scopus database.

The relationship between urban planning and citizen well-being started in 1982, with increasing interest until 2024. Such an increase has generated a trend in revealing the effectiveness of urban planning implemented by local governments on citizen satisfaction over the last decade for social demands increasingly noticeable as of 2019 ( Figure 2).

**Figure 3.**
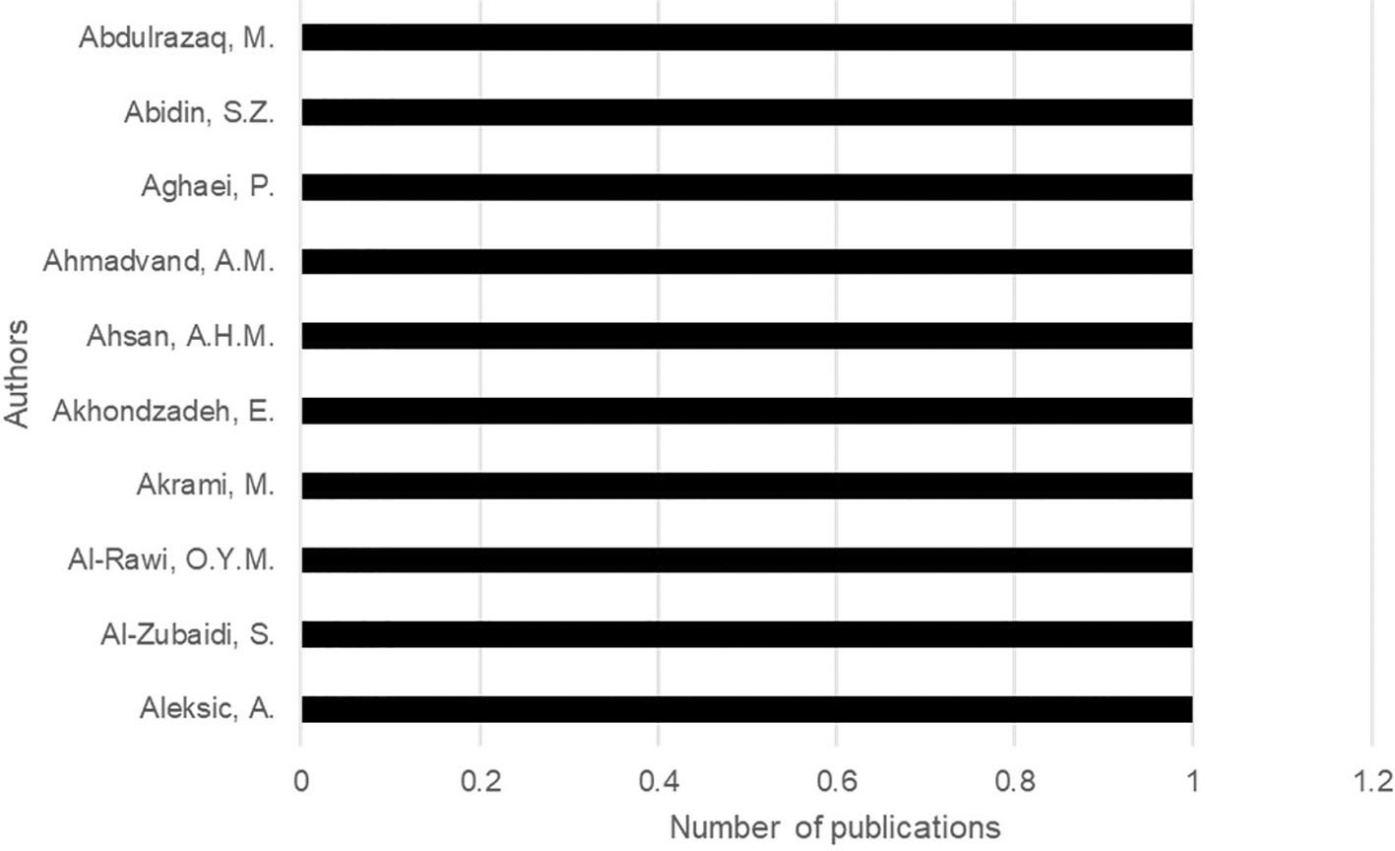
Main authors contributing to the collection. *Note.* Retrieved from Scopus database.

The most relevant authors are Abdulrazaq from Baghdad University in Baghdad, Abidin from Teknologi Mara University in Malaysia, Aghaei from Isfahan University of Technology in Australia ( Figure 3).

**Figure 4.**
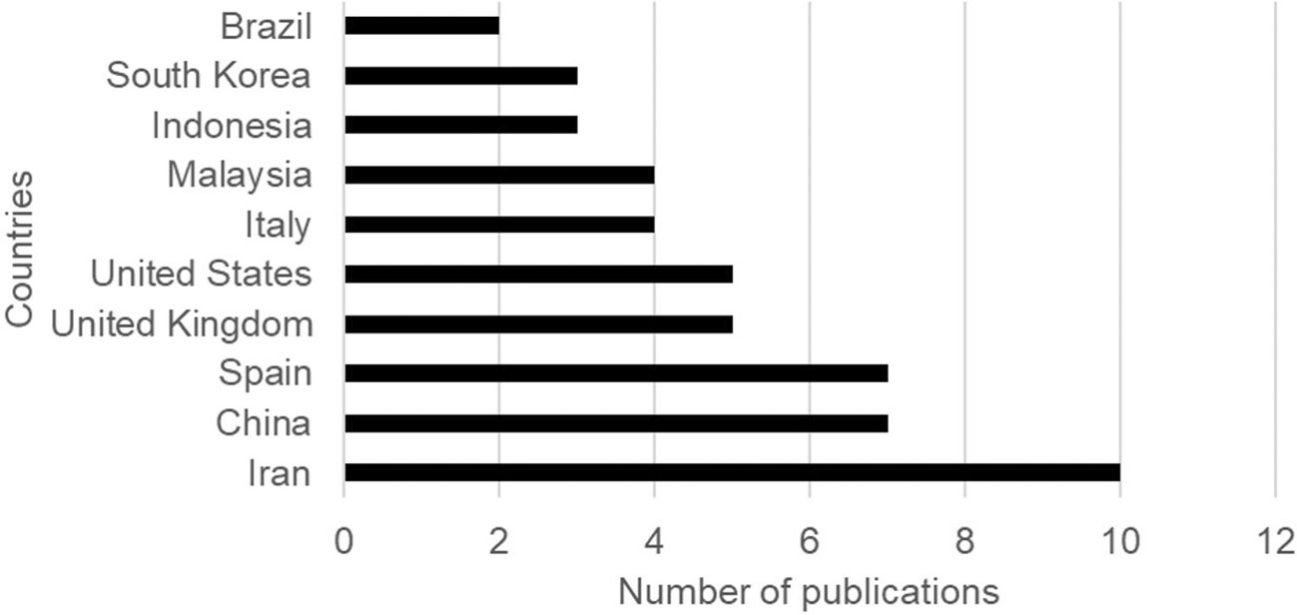
Publications by country on urban planning and citizen well-being. *Note.* Retrieved from Scopus database.

In the analysis of publications by country, the largest contributions to the literature are concentrated in the countries of Iran (13%), China (9%), Spain (9%), the United Kingdom (6%), the United States (6%) and Italy (5%) on the relationship between urban planning and citizen well-being ( Figure 4).

For the analysis of the authors’ contribution we use Lotka’s Law, a mathematical regularity proposed by Alfred J. Lotka in 1926, which describes the frequency of publication in a scientific field. Considering this law, a small group of authors with the highest production generates most of the scientific literature in a given area, being 100% who produce an article.

Bradford’s law, also called the law of dispersion of scientific literature, proposed by Samuel C. Bradford in 1934, describes the distribution of articles on a topic in scientific journals. It highlights the existence of a core group of journals that concentrate most of the relevant articles on a specific topic, which makes them the main sources of expertise ( Table 2).

**Table 2.** Bradford Law.

Magazine	Ranking	Frequency	Cumulative frequency	Zone
INTERNATIONAL JOURNAL OF HUMAN CAPITAL IN URBAN MANAGEMENT	1	2	2	Zone 1
IOP CONFERENCE SERIES: EARTH AND ENVIRONMENTAL SCIENCE	2	2	4	Zone 1
JOURNAL OF ENVIRONMENTAL ENGINEERING AND LANDSCAPE MANAGEMENT	3	2	6	Zone 1
PLOS ONE	4	2	8	Zone 1
17TH ITS WORLD CONGRESS	5	1	9	Zone 1
2020 INTERNATIONAL WIRELESS COMMUNICATIONS AND MOBILE COMPUTING, IWCMC 2020	6	1	10	Zone 1
2022 IEEE INTERNATIONAL CONFERENCE ON AUTOMATIC CONTROL AND INTELLIGENT SYSTEMS, I2CACIS 2022 - PROCEEDINGS	7	1	11	Zone 1
2024 47TH ICT AND ELECTRONICS CONVENTION, MIPRO 2024 - PROCEEDINGS	8	1	12	Zone 1
ADVANCES IN SCIENCE, TECHNOLOGY AND INNOVATION	9	1	13	Zone 1
AIP CONFERENCE PROCEEDINGS	10	1	14	Zone 1

Note: Obtaining the Scopus database from Bibliometrix.

**Figure 5.**
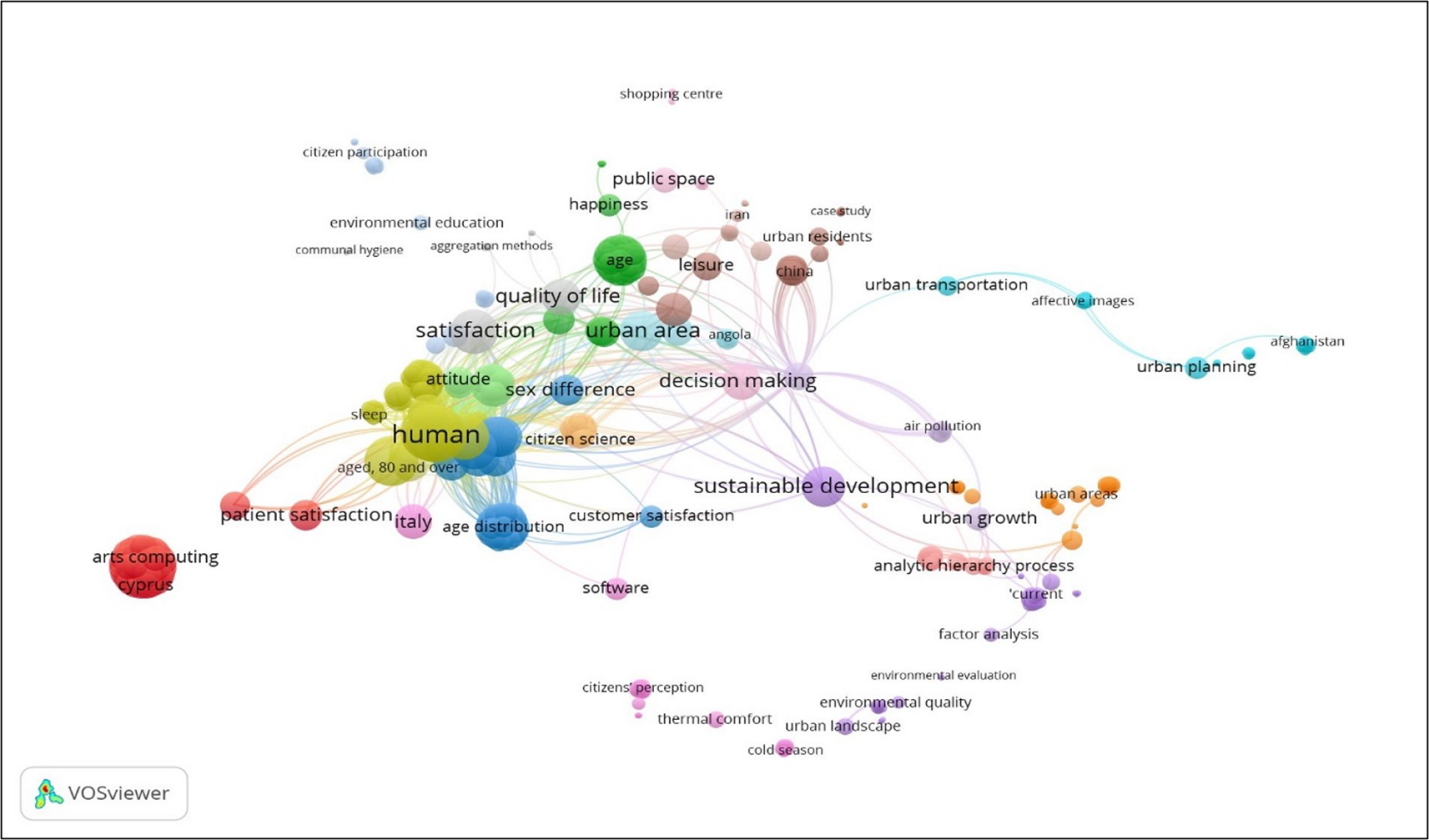
Semantic map of the relationship between urban planning implemented by local governments and citizen satisfaction. *Note.* Obtained from the open source programme VOSviewer, with metadata from Scopus.

For the state of the art of the relationship between urban planning implemented by local governments and citizen satisfaction, a semantic analysis is presented based on the key terms used in the research and the relationships between their authors, journals, sponsors, institutional affiliations and other metadata, which in an underlying way generate a dynamic in a research ecosystem and, In the analysis it is evident that both variables have a close relationship, where at the same time the clusters indicate a closeness between variables such as sustainable development, happiness, public space, urban growth, urban areas, satisfied customers, urban residents, among others. The construction of semantic maps uses the arguments proposed by Zipf’s law, also known as the law of least effort, which is an empirical law proposed by the American linguist George Kingsley Zipf in 1949. This law refers to the frequency of occurrence of words in a text or linguistic corpus ( Figure 5).

The contribution of the relationship between urban planning implemented by local governments and citizen satisfaction is agreed to have a positive effect when considering a planned city that generates effective conditions for improved development outcomes ( Liu et al., 2022; Orr & West, 2002; Sánchez et al., 2021).

The extant literature suggests a positive correlation between planning instruments based on land use, mobility, public space, smart arrangements, and citizen satisfaction when three mechanisms converge: accessibility, quality of the environment, and legitimacy involving participation and transparency. It is important to note that these effects are context-dependent and therefore should not be interpreted as universal.

Research indicates that, in the context of political-institutional constraints or designated policy frameworks, urban planning initiatives may not necessarily result in enhanced satisfaction levels. In some cases, these initiatives can even serve to exacerbate existing inequalities. This observation highlights the necessity for distributive safeguards and a territory-sensitive design approach to ensure the effective and equitable implementation of urban planning policies.

It is important to acknowledge that levels of dissatisfaction have the capacity to precede and precipitate meticulous planning reforms within governmental agendas, thereby introducing endogeneity into observational estimates. Consequently, the observed patterns are interpreted as associations rather than definitive causal evidence. It is recommended that future research employs time series, differences in differences, discontinuity designs or plausible instruments.

The efficacy of planning is predicated on local administrative capacity, which is influenced by resources, data and execution, as well as the political regime and degree of openness, informality and land tenure regime, territorial equity and urban morphology, which engender inequitable results.

The systematic literature review has identified three main building blocks in the contribution of urban planning to citizen satisfaction. These blocks highlight the importance of planning effectiveness in providing a solid basis for future research into the design of local public policies, as shown in Table 3.

**Table 3.** Outcome of the individual studies.

Authors	Contribution block	Urban planning guidelines
Aleksic et al. (2019); Colderley (1999); Croese, S. and Pitcher, M (2019); Fang et al. (2020)	**Urban planning as a catalyst for the impact of citizen satisfaction.** Planning has been a relevant contribution to local policy formulation considering city master plans and use plans; being crucial for quality of life; having a new approach to Neoliberalism.	Development of local policies based on the neoliberal approach to smart urban development.
Jackson (2018); Lo Piccolo (2017); Niu et al. (2017); Orr, M and West, D (2002); Sánchez et al. (2021); Santos et al. (2007)	**Theoretical input for urban planning.** Theoretical input is based on Neoliberalism for urban and regional planning; as well as modernist planning and a new multidimensional construct with a multidimensional nature for urban revitalisation considering synthetic indicators for quality of life and considering a complementary approach.	The various theoretical contributions contextualise a modernist planning based on a multidimensional aspect to address quality of life for modern well-being.
Silva (2020); Silva (2016); Stankovic et al. (2017); Ulloa (2016); Varró, K and Szalai A (2022); Wang et al.(2023); Wang, Z and Zhong, Y. (2020); Yermachenko, V. (2023); Xue, J and Kębłowski, W (2022).	**Smart planning for citizen satisfaction.** Planning tools and effective transformation of the territory based on smart planning enable the development of a smart city for spatial planning system in co-constitutive performance for centralised urban development **.** Emphasising smart governance for socio-spatial development, considering a socio-ecological transformation to contribute to optimal urbanism and well-being of life.	Deepen the design and implementation of public governance reforms aimed at optimising the management of urban planning at the local level for smart urban city development.

Note: Obtaining the analysed database.

The first block entitled “Urban planning as a catalyst for the impact of citizen satisfaction”, studies agree that planning is a relevant input for the formulation of local policies to improve the quality of life of citizens with a neoliberal approach ( Aleksic et al., 2019; Colderley, 1999; Croese & Pitcher, 2019; Fang et al., 2020).

Planning is not merely regarded as a list of instruments; rather, it is approached as an intermediate causal function that converts public inputs into results perceived by citizens. The following characteristics are detailed ( Croese & Pitcher, 2019; Fang et al., 2020):

• **Allocation and accessibility**: planning redistributes land use, mobility, and services, reducing access times and costs;
• **Environmental quality and public space**: design and regulation increase livability and well-being;
• **Co-production and trust**: participatory planning aligns expectations and reinforces legitimacy, increasing satisfaction.

The second section, entitled “Theoretical contributions to urban planning”, reveals that the authors’ relevant contributions focus on highlighting the trends of modernism (spatial efficiency), capacity and well-being approaches (multidimensional quality of life), collaborative governance (legitimacy and co-production), urban resilience (risk management), and smart cities (digital infrastructure and data). This category facilitates a critical evaluation of the doctrinal frameworks that have guided planning practice, encompassing modernist planning with its emphasis on spatial planning, as well as contemporary trends that incorporate social, environmental, and participatory dimensions. This debate is not incidental; rather, it provides the conceptual foundation for the transition towards more inclusive and resilient planning models, which are designed to respond to the challenges of contemporary cities ( Jackson, 2018; Lo Piccolo, 2017; Niu et al., 2017; Orr & West, 2002; Sánchez et al., 2021; Santos et al., 2007).

The third block “Smart planning for citizen satisfaction” involves the authors’ consensus that planning tools serve for the effective transformation of the territory considering smart planning for the development of a spatial planning system where smart governance stands out, which is transformed into optimal urbanism and a timely and development-generating socio-spatial development.

The concept of smart planning synthesises prior learning and projects it onto contemporary urban governance, in which technology, citizen participation, and sustainability are converging pillars. Research has demonstrated that smart tools (big data, digital governance, sustainable mobility systems) become operational expressions of planning effectiveness, directly linking local policies with perceptions of citizen satisfaction in complex urban environments.

In this context, the effectiveness of urban planning implemented by local governments on citizen satisfaction is still deficient, given that in the multilevel and smart governance environment a multidimensional approach is required where the contribution to quality of life allows inferring the new modernist planning approach to achieve the development of cities in line with improved policy making and leads to the establishment of new and timely approaches to urban planning based on new public governance.

The contribution of this systematic review therefore lies in its systematisation of these three categories within a comparative and relational framework, where each block reinforces the others: planning as a catalyst explains the mechanism, theoretical contributions provide the conceptual basis, and smart planning demonstrates contemporary application and its measurable effects. This assembly facilitates progression from a state of mere description to an interpretive model that establishes a connection between theory, practice, and citizen perception. It demonstrates that urban planning not only structures space but also has the capacity to influence citizen satisfaction and well-being.

In this regard, we maintain that the article makes a significant contribution on two levels: (i) at the academic level, by integrating three disparate analytical traditions into a coherent framework, and (ii) at the practical level, by offering local governments a roadmap for designing urban policies that translate planning into tangible well-being for citizens.

Therefore, given the context described above, addressing the link between urban planning and citizen satisfaction would give rise to new related categories: (i) territorial equity, (ii) local institutional capacity, (iii) urban density and morphology, (iv) land informality, and (v) quality of participation. This approach provides an explanatory model that connects mechanisms with theories and operationalization, showing how and under what conditions urban planning increases citizen satisfaction, and when it may not.

It is acknowledged that there are limitations in the evidence, including heterogeneity, publication bias, and the use of proxy markers of satisfaction. In light of these limitations, the following lines of advance are proposed: meta-evaluations with comparable indicators and quasi-experimental designs, and high-frequency metrics of citizen experience.

## Discussion

The contribution of the research reveals the synthesis and systematisation of the state of the art evidence on the positive relationship of local government urban planning on citizen satisfaction. A detailed review of the literature has identified significant convergences in the empirical findings of various researchers in different geographical contexts. This exhaustive literature review has allowed us to identify convergence in the empirical findings that originate from different geographical contexts. This review is grouped into contributions from three main thematic blocks, which involves facilitating the understanding of the diverse theoretical approach linked to sustainable development, happiness, public space, urban growth, urban areas, satisfied customers and urban residents, generating greater effectiveness in the territorial environment.

The results of the systematic review demonstrate that urban planning has a significant impact on citizen satisfaction; however, the findings indicate considerable heterogeneity, which necessitates a cautious interpretation of the results. The extant literature does not converge on a single explanatory model, but rather offers divergent evidence that must be critically addressed. The positive correlation between planning and satisfaction is contingent on the mechanisms that are activated, including accessibility, environmental quality, and trust in management. The governance context in which the planning is implemented also exerts influence.

Consequently, certain studies have documented enhancements in satisfaction associated with sustainable mobility interventions or green spaces ( Shahsavar et al., 2022; Wang et al., 2023; Samavati et al., 2024). Conversely, other studies have emphasised either no effects or adverse effects in the context of urban renewal processes connected with gentrification, displacement of vulnerable populations, or elevated cost of living.

The analysis highlights a series of conceptual contradictions that must be critically acknowledged. Firstly, a tension exists between the concepts of citizen participation and technocracy. While certain studies have emphasised that participation strengthens legitimacy and social satisfaction, others have highlighted that consultative processes without effective impact can lead to frustration and mistrust among the population. A further dilemma exists between smart governance and equity. While smart city initiatives can enhance management efficiency, they can also perpetuate inequalities if digital divides remain unaddressed and spatial justice criteria are not implemented. In a similar fashion, the concept of urban resilience frequently finds itself in conflict with austerity policies. This is due to the fact that interventions which are designed to enhance response capacity may coincide with fiscal reductions that result in costs being transferred to the most vulnerable communities. This, in turn, has the effect of limiting the positive effects on citizen satisfaction. Consequently, modernist urban planning practices are characterised by a tension with urban proximities, stemming from an emphasis on spatial efficiency and rigid planning, which contrasts with everyday practices and residential preferences. This discord leads to a less favourable perception among citizens.

The contribution of the positive and substantive relationship between urban planning and citizen satisfaction, the systematic review of the literature reveals a widespread consensus among researchers, which gives rise to the importance of urban planning. In line with this result, participatory planning has emerged as a key trend, promoting the inclusion of diverse voices in the decision-making process, which translates into greater legitimacy and effectiveness of urban policies ( Hamdouch & Galvan, 2019). Likewise Shahsavar et al. (2022) engages resilience-based planning, which focuses on the capacity of cities to adapt to and recover from crises and disasters, whether natural or human-induced. This approach has become increasingly crucial in a context of climate change and rapid urbanisation, where cities must be able to cope with unpredictable challenges. The literature also highlights the importance of technology and innovation in urban planning, with a growing interest in the use of digital tools and big data to improve urban management and citizen participation ( Wang et al., 2023).

In this review, the three categories are integrated into an explanatory framework based on mechanisms and conditions of validity. Planning as a catalyst acts through (i) allocation and accessibility, (ii) quality of public space, and (iii) co-production and trust; these pathways are supported by a pluralistic theoretical corpus—modernism, well-being, capabilities, collaborative governance, resilience, and smart cities—that explains why the mechanisms work and when they fail. Smart planning operationalizes and evaluates this framework with traceable metrics, learning loops, and evaluative designs that link outputs with satisfaction outcomes. By defining “neoliberalism” and “modernist planning” as operational constructs and by specifying moderators (territorial equity, institutional capacity, morphology, informality, and quality of participation), the model moves from descriptive synthesis to a testable proposition about the effectiveness of planning in increasing citizen satisfaction.

A frequently overlooked aspect in the extant literature pertains to the phenomenon of reverse causality, whereby citizen dissatisfaction may act as a catalyst for planning reforms, thereby introducing risks of endogeneity in observational studies. This finding indicates that the observed patterns should be interpreted as contextual associations rather than definitive causal relationships. Consequently, structural governance constraints, such as inadequate institutional capacity, land informality, or the political capture of planning instruments, impede the efficacy of interventions and elucidate why, in certain contexts, the advantages are negligible or non-existent.

This analysis serves to both reaffirm the consensus on the importance of participation, resilience, and smart governance, and to incorporate the conditions of validity under which these approaches produce citizen satisfaction. Rather than proposing a universal positive effect, the evidence suggests a conditional model: the satisfaction of residents is increased by urban planning if and only if improvements in accessibility, environmental quality, and effective co-production processes are simultaneously implemented, and if institutional capacities exist to maintain the changes.

The main limitation of systematic literature reviews is the possible omission of relevant studies not indexed in the databases used. Although the Scopus, WOS and Dimensions databases with wide coverage were used, future reviews could consider incorporating other sources. Since it is important that this is a multidimensional phenomenon, the keyword search may miss some studies that use different terminology.

In terms of future research perspectives, it is recommended to deepen empirical studies, possibly quantitative, that examine the relative weight of the different dimensions of urban planning that contribute to citizen satisfaction and assess the effectiveness of urban planning in the territories.

## Conclusions

The systematic review undertaken in this study identifies that the relationship between urban planning and citizen satisfaction is an emerging field, with significant conceptual advances, but still characterised by fragmented and contextually dependent evidence. The set of 22 selected studies provides consistent evidence that certain planning approaches – those that incorporate sustainability, accessibility, and participation – tend to be associated with more favourable perceptions of citizen well-being. However, it is important to note that these results should not be interpreted as solid and generalizable empirical support. Rather, they should be considered as indications of conditional patterns that require further verification in different urban contexts.

The findings demonstrate that strategic planning can function as a catalyst for perceived enhancements in quality of life when it translates territorial objectives into tangible benefits for citizens; that theoretical contributions introduce doctrinal diversity to the debate, encompassing modernist approaches and critiques of neoliberalism, as well as perspectives focused on resilience and spatial justice; and that intelligent planning offers novel instruments for managing urban complexity, although with the risk of reproducing inequalities if equity criteria are not applied. Instead of demonstrating a definitive consensus, these three categories reflect the heterogeneity of existing approaches and the ongoing debates within the relevant literature.

The bibliometric analysis revealed research clusters and thematic networks that reflect the convergence of debates on participation, digital governance, and urban resilience. However, it is important to note that these groupings should not be extrapolated into definitive theoretical conclusions. Rather, they should be understood as a starting point to guide future research. The results obtained reveal discrepancies, conceptual inconsistencies and the necessity for comparative studies in order to elucidate the conditions under which such practices can effectively contribute to enhancing citizen satisfaction. Rather than confirming consensus, these results indicate a need for further investigation.

In addition, the scientific evidence presented corroborates the hypothesis that planning has been employed in a beneficial and efficient manner in various local governments within the framework of modernist planning. This approach encompasses a multidimensional perspective on the development of intelligent planning, which facilitates multilevel governance for sustainable development with an emphasis on economic and social development objectives. This systematic review provides a solid foundation for future research and the design of local public policies aimed at strengthening governance to optimise the use of public resources.

The principal contribution of this work is not to affirm the existence of a consolidated theory, but to underscore the necessity for expansion and deepening of empirical evidence, particularly in diverse contexts such as Latin America, where urban planning confronts structural challenges that differ from those documented in developed countries. It is recommended that future research adopt more integrative analytical frameworks capable of capturing the tensions between participation and technocracy, efficiency and equity, or resilience and austerity. This would provide a more robust empirical base to support coherent theoretical and policy proposals.

### Ethics and consent

Ethics and consent were not required for the performed study.

## Data Availability

Zenodo: Urban planning effectiveness and citizen satisfaction. A systematic literature review. Version 4. https://doi.org/10.5281/zenodo.17308186 ( Llonto et al., 2025). The project contains the following underlying data:
• Author Contributions.xlsx (Results of the analysis of the contributions by author and by blocks). • Bibliometric figures.xlsx (Results of the data in tables and figures obtained from the databases). • Dimensions Data.xlsx (Raw data from the Dimensions database). • General data of the RSL process.xlsx (Results of the systematic literature review from Scopus, Web Of Science, Dimensions database). • ResEB.xlsx (Matrix of development of scientific information search equations). • Scopus database analysis.xlsx (Result of the search equation by affiliation, year, country, subject area, type of document and author). • Scopus database.xlsx (Processed data from the Scopus database). • Documents from the Data RSL on Urban Planning and Satisfaction (Final list of selected documents). Zenodo: Urban planning effectiveness and citizen satisfaction. A systematic literature review. Version 4. https://doi.org/10.5281/zenodo.17308186 ( Llonto et al., 2025). This project contains the following extended data:
• Supplementary Figure 1. (Diagram of the Prisma 2020 Flow Chart for item selection). • Supplementary Figure 2. (Developments in publications on urban planning and citizen welfare). • Supplementary Figure 3. (Main authors contributing to the collection) • Supplementary Figure 4. (Publications by country on urban planning and citizen well-being). • Supplementary Figure 5: (Semantic map of the relationship between urban planning implemented by local governments and citizen satisfaction). Zenodo: PRISMA checklist for ‘Urban planning effectiveness and citizen satisfaction. A systematic literature review’. Version 4. https://doi.org/10.5281/zenodo.17308186 ( Llonto et al., 2025). Data are available under the terms of the Creative Commons Zero “No rights reserved” data waiver (CC0 1.0 Public domain dedication).
