# Supplementary material for: Urban planning effectiveness and citizen satisfaction. A systematic literature review
Source: F1000Res. 2025 Oct 16;13:1330. Originally published 2024 Nov 6. [Version 2] doi: 10.12688/f1000research.157550.2 (PMC12640496; doi:10.12688/f1000research.157550.2)
Supplement: Peer review history

# Reviewer report: Reviewer response for version 2

**DOI:** 10.5256/f1000research.188569.r426456

**Published:** 2025-11-22

**Authors:** Abdullah Saeed Karban

**Affiliations:** 1 Umm Al-Qura University, Makkah, Saudi Arabia

The manuscript presents a systematic literature review exploring how urban planning implemented by local governments affects citizen satisfaction. Using PRISMA 2020 guidelines, the authors synthesize evidence from Scopus, Web of Science, and Dimensions, narrowing 234 initial records to 22 included studies. The review combines qualitative synthesis,and thematic grouping into three concepts including: 

1. **Urban planning as a catalyst for citizen satisfaction**,
2. **Theoretical contributions to urban planning**, and
3. **Smart planning and governance for satisfaction**.

The authors conclude that planning influences satisfaction primarily when it improves accessibility, environmental quality, and citizen participation, though contextual differences and governance capacity can moderate outcomes.

 The manuscript is valuable but requires to improve conceptual clarity, methodological detail, and the accuracy of conclusions as show in the following suggestions: 

**Strengthen the rationale.**

 The justification for the study is unclear and fragmented. Add a concise *Theoretical Framework* section defining key concepts such as neoliberalism, modernist planning, and smart governance.

**Improve methodological transparency.**

 The methods lack sufficient detail for reproducibility. Clearly describe the quality assessment tool, provide scoring examples, justify the 2.3 threshold, state exact search dates, explain reviewer calibration, and describe how disagreements were resolved. Acknowledge the previously incorrect Web of Science search string.

**Revise the conclusions.**

 Avoid overgeneralized statements. Use more cautious language and incorporate contradictory findings and the conceptual tensions discussed in the manuscript.

Are the rationale for, and objectives of, the Systematic Review clearly stated?

Partly

Is the statistical analysis and its interpretation appropriate?

Not applicable

If this is a Living Systematic Review, is the ‘living’ method appropriate and is the search schedule clearly defined and justified? (‘Living Systematic Review’ or a variation of this term should be included in the title.)

Not applicable

Are sufficient details of the methods and analysis provided to allow replication by others?

Partly

Are the conclusions drawn adequately supported by the results presented in the review?

Partly

Reviewer Expertise:

I am an architect and urban planner with expertise in urban and regional planning, neighborhood sustainability assessment, and human-centered design research. My work focuses on spatial behavior, wayfinding systems, pedestrian movement, and evidence-based planning evaluation. This background enables me to critically assess the conceptual, methodological, and practical aspects of research related to urban planning effectiveness and citizen satisfaction.

I confirm that I have read this submission and believe that I have an appropriate level of expertise to confirm that it is of an acceptable scientific standard, however I have significant reservations, as outlined above.

# Reviewer report: Reviewer response for version 2

**DOI:** 10.5256/f1000research.188569.r424736

**Published:** 2025-10-25

**Authors:** Marco Agustin Arbulu Ballesteros

**Affiliations:** 1 Universidad de San Martín de Porres, Molina, Lima, Peru

After carefully reviewing the corrected version of the manuscript, I confirm my approval of the authors' response to the observations previously raised. The authors have thoroughly and satisfactorily addressed each of the comments and suggestions made during the review process, incorporating the necessary modifications that have significantly strengthened the quality and clarity of the work. The corrections demonstrate the researchers' commitment to academic rigor and their ability to effectively integrate constructive feedback. I commend the authors for their dedication and professionalism in refining this valuable contribution to the scientific literature.

Are the rationale for, and objectives of, the Systematic Review clearly stated?

Yes

Is the statistical analysis and its interpretation appropriate?

Yes

If this is a Living Systematic Review, is the ‘living’ method appropriate and is the search schedule clearly defined and justified? (‘Living Systematic Review’ or a variation of this term should be included in the title.)

Yes

Are sufficient details of the methods and analysis provided to allow replication by others?

Partly

Are the conclusions drawn adequately supported by the results presented in the review?

Yes

Reviewer Expertise:

Management, human behavior and consumer behavior.Gestión, comportamiento del ser humano y comportamiento del consumidor.

I confirm that I have read this submission and believe that I have an appropriate level of expertise to confirm that it is of an acceptable scientific standard.

# Reviewer report: Reviewer response for version 1

**DOI:** 10.5256/f1000research.173008.r412273

**Published:** 2025-09-25

**Authors:** Marco Agustin Arbulu Ballesteros

**Affiliations:** 1 Universidad de San Martín de Porres, Molina, Lima, Peru

**1. Conceptual Clarity and Theoretical Framing:**

- **Critique:** The central concepts of "neoliberalism" and "modernist planning" are used repeatedly but are not adequately defined or problematized. They are presented as accepted frameworks without engaging with the critical debates that surround them in urban studies literature. This gives the analysis a somewhat superficial and asserted feel, rather than one that is critically examined.
- **Recommendation:**
  - **Add a dedicated section** early in the paper (e.g., in the introduction or a new "Theoretical Framework" section) that clearly defines what you mean by "neoliberal approach" and "modernist planning" in the context of your review.
  - **Briefly acknowledge the critiques** of these approaches (e.g., neoliberal planning's potential to exacerbate inequality; modernist planning's potential to be top-down and ignore local context). This will demonstrate a deeper engagement with the field.
  - **Justify why these are useful lenses** for analyzing the relationship between planning and satisfaction, rather than simply presenting them as given.

**2. Methodological Rigor and Transparency:**

- **Critique A (Search Strategy Error):** There is a critical inconsistency in the search protocol. The query for Web of Science (WOS) is reported as WOS: (“economy” AND “crime” AND “Covid-19”), which is completely unrelated to the research topic. This major error undermines the reliability of the entire systematic search process.
- **Recommendation A:**
  - **Correct the search string** for WOS to align with the topic. It should be similar to those used for Scopus and Dimensions, for example: TS=(("urban planning" OR "urban order") AND ("citizen satisfaction")).
  - **Re-run the search** with the correct query and report the new numbers. Update the PRISMA flowchart and the entire results section accordingly. This is essential for the study's validity.
- **Critique B (Quality Assessment & Risk of Bias):** The methodology mentions a "traffic lights" figure for risk assessment but provides no explanation of what this is, what criteria were used, or how it was applied. A systematic review must include a clear and replicable process for assessing the quality and potential bias of the included studies.
- **Recommendation B:**
  - **Describe the quality assessment tool** in detail. What was the "traffic light" system based on? (e.g., the Joanna Briggs Institute (JBI) critical appraisal checklists? Custom criteria?).
  - **Present the results** of this assessment, perhaps in a supplementary table. This allows readers to gauge the strength of the evidence you are synthesizing.
  - **Explain how the quality assessment** informed your analysis and conclusions (e.g., were lower-quality studies given less weight?).

**3. Depth of Analysis and Interpretation of Findings:**

- **Critique A (Over-Generalization):** The conclusion that there is "strong empirical support" and a "general consensus" is not fully supported by the evidence presented. With a final set of only 22 studies from specific geographical contexts (predominantly Europe and Asia), the findings are not easily generalizable. The review does not adequately engage with contradictory evidence or the context-dependent nature of planning outcomes.
- **Recommendation A:**
  - **Tone down the language** of certainty. Use phrases like "the reviewed literature suggests..." or "evidence from the included studies points to a potential relationship...".
  - **Add a subsection on "Limitations of the Evidence"** within the discussion. Explicitly discuss the geographical bias, the small sample size of studies, and the methodological variety that may prevent firm conclusions.
  - **Actively look for and discuss** any studies in your sample that reported mixed, negative, or null results regarding the planning-satisfaction link.
- **Critique B (Lack of Critical Engagement):** The discussion primarily restates the findings and agrees with the consensus in the literature. It does not critically interrogate *why* results might differ, explore potential reverse causality (e.g., does citizen dissatisfaction drive planning reforms?), or discuss the structural and political barriers that limit planning effectiveness in real-world contexts.
- **Recommendation B:**
  - **Deepen the discussion section** by synthesizing across the three blocks to identify tensions, gaps, and unanswered questions. For example: "While Block 3 promotes smart governance, several studies in Block 1 highlight implementation deficits in local governments. This suggests that technological solutions may be hampered by existing governance challenges."
  - **Consider alternative explanations** for the findings and explicitly state them.

**4. Scope and Literature Base:**

- **Critique:** The literature base, as presented, has a limited scope. The reviewer rightly points out that the study could be enriched by engaging with related frameworks like the Social-Ecological System (SES) framework, which is highly relevant to understanding participatory governance and resource management in urban settings (as seen in the suggested references by Shi & Ling, 2025).
- **Recommendation:**
  - **Acknowledge this as a limitation** of the current review's scope.
  - **In the "Future Research" section,** explicitly propose examining the relationship between urban planning and satisfaction through other theoretical lenses, such as the SES framework or collective action theory. This demonstrates a broader understanding of the field.

**5. Conclusion and Contribution:**

- **Critique:** The conclusions overstate the findings and do not clearly articulate the specific, novel contribution this review makes to the existing body of knowledge.
- **Recommendation:**
  - **Ensure conclusions are a direct reflection** of the results and discussion, acknowledging the limitations.
  - **Clearly state the review's contribution.** Is it the three-block synthesis model? The identification of a specific gap? Reframe the conclusion to highlight what new understanding your review provides that wasn't already known.

Are the rationale for, and objectives of, the Systematic Review clearly stated?

Yes

Is the statistical analysis and its interpretation appropriate?

Yes

If this is a Living Systematic Review, is the ‘living’ method appropriate and is the search schedule clearly defined and justified? (‘Living Systematic Review’ or a variation of this term should be included in the title.)

Yes

Are sufficient details of the methods and analysis provided to allow replication by others?

Partly

Are the conclusions drawn adequately supported by the results presented in the review?

Yes

Reviewer Expertise:

Management, human behavior and consumer behavior.Gestión, comportamiento del ser humano y comportamiento del consumidor.

I confirm that I have read this submission and believe that I have an appropriate level of expertise to confirm that it is of an acceptable scientific standard, however I have significant reservations, as outlined above.

# Reviewer report: Reviewer response for version 1

**DOI:** 10.5256/f1000research.173008.r399902

**Published:** 2025-09-04

**Authors:** Gabriel H T Ling

**Affiliations:** 1 Department of Urban and Regional Planning, Universiti Teknologi Malaysia, Skudai, Johor, Malaysia

The paper addresses an important theme the effectiveness of urban planning on citizen satisfaction but its overall contribution is modest and its execution problematic. While it usefully synthesises evidence into three conceptual blocks (planning as a catalyst, theoretical contributions, and smart planning), these categories are overly broad and descriptive, lacking deeper theoretical integration or critical debate; moreover, the repeated emphasis on “neoliberalism” and “modernist planning” is asserted without adequate conceptual justification, making the framing appear forced.

 The literature review is current and touches on relevant debates around smart cities, participation, resilience, and sustainability, yet it remains superficial: it conflates satisfaction, well-being, and happiness without distinction, over-relies on general citations, and pays little attention to geographical diversity beyond a handful of cases, resulting in a Euro- and Asia-centric scope.

 Methodologically, the systematic review is weakened by inconsistencies in database queries (e.g., WoS query terms unrelated to the research focus), a limited final dataset of only 22 studies, and vague quality assessment procedures. 

 What does this mean: "For the analysis of the risk assessment, use is made of the figure of traffic lights, taking a critical view that contributes to

 the research"

 Findings are over-generalised: the paper asserts a strong positive relationship between urban planning and citizen satisfaction, while glossing over contradictory or context-dependent evidence and ignoring issues such as reverse causality (citizen dissatisfaction driving planning reforms) or structural limitations in governance.

 The discussion does not critically engage with the heterogeneity of findings or interrogate tensions within the literature, instead restating consensus positions on participation, smart governance, and resilience without considering competing or dissenting perspectives.

 The conclusion, while neatly summarised, significantly overstates the evidence base by claiming “strong empirical support” from a very small and heterogeneous set of studies, and by extrapolating bibliometric clustering into substantive theoretical claims. Overall, the manuscript is clearly written and well structured, but it remains descriptive, lacks critical depth, and does not convincingly advance knowledge in the field; to be suitable for publication it would require major revisions, including a more robust and transparent search and appraisal strategy, sharper conceptual clarity, genuine engagement with conflicting evidence, and more cautious, evidence-aligned conclusions.

 To enrich the study's literature, authors can refer to the following articles that focus on smart cities and public participation, social ecological system in resource planning and management:

Smart cities and public participation: a bibliometric analysis and conceptualization of the social-ecological system framework | Open House International | Emerald Publishing (Refer 1)

A systematic review of factors influencing self-governed public open spaces: insights from social-ecological system framework and collective action | Open House International | Emerald Publishing (Refer2) 

 -

Are the rationale for, and objectives of, the Systematic Review clearly stated?

Yes

Is the statistical analysis and its interpretation appropriate?

Not applicable

If this is a Living Systematic Review, is the ‘living’ method appropriate and is the search schedule clearly defined and justified? (‘Living Systematic Review’ or a variation of this term should be included in the title.)

No

Are sufficient details of the methods and analysis provided to allow replication by others?

Yes

Are the conclusions drawn adequately supported by the results presented in the review?

Partly

Reviewer Expertise:

Urban planning, social ecological system, Housing, climate change

I confirm that I have read this submission and believe that I have an appropriate level of expertise to confirm that it is of an acceptable scientific standard, however I have significant reservations, as outlined above.

## References

[1] : Smart cities and public participation: a bibliometric analysis and conceptualization of the social-ecological system framework. *Open House International* .2025; 10.1108/OHI-11-2024-0372 10.1108/OHI-11-2024-0372

[2] : A systematic review of factors influencing self-governed public open spaces: insights from social-ecological system framework and collective action. *Open House International* .2025;50(1) : 10.1108/OHI-11-2023-0269 20-39 10.1108/OHI-11-2023-0269
